# Cell sources and engineering strategies for cultured meat production: a critical comparative review

**DOI:** 10.3389/fnut.2026.1791530

**Published:** 2026-06-02

**Authors:** Dominika Domagała, Aleksandra Partyńska, Krzysztof Data, Piotr Paweł Chmielewski, Julia Niebora, Magdalena Kulus, Jakub Kulus, Artur Bryja, Dorota Bukowska, Paweł Antosik, Maciej Zabel, Piotr Dziȩgiel, Paul Mozdziak, Bartosz Kempisty

**Affiliations:** 1 Department of Human Morphology and Embryology, Division of Anatomy, Faculty of Medicine, Wroclaw Medical University, Wroclaw, Poland; 2 Department of Human Morphology and Embryology, Division of Histology and Embryology, Wroclaw Medical University, Wroclaw, Poland; 3 Department of Veterinary Surgery, Institute of Veterinary Medicine, Nicolaus Copernicus University in Torun, Torun, Poland; 4 Department of Diagnostics and Clinical Sciences, Institute of Veterinary Medicine, Nicolaus Copernicus University in Torun, Torun, Poland; 5 Division of Anatomy and Histology, University of Zielona Góra, Zielona Góra, Poland; 6 Department of Human Biology, Faculty of Physiotherapy, Wroclaw University of Health and Sport Sciences, Wroclaw, Poland; 7 Graduate Physiology Faculty, North Carolina State University, Raleigh, NC, United States; 8 Prestage Department of Poultry Science, North Carolina State University, Raleigh, NC, United States; 9 Department of Obstetrics and Gynecology, University Hospital and Masaryk University, Brno, Czechia

**Keywords:** bioreactors, cellular agriculture, cultured meat, meat alternatives, satellite cells, scaffold biomaterials, serum-free media

## Abstract

Meat production and ethical concerns related to animal welfare have led to an increase in research into the creation of conventional meat. These technologies rely on the isolation, multiplication, and controlled differentiation of animal cells, which can potentially reduce negative environmental impact while maintaining comparable nutritional and sensory properties. The aim of this study is to comprehensively analyze and compare selected cell types used in the production of cultured meat, such as fibroblasts, satellite cells, adipocytes, and pluripotent cells: embryonic stem cells (ESCs) and induced pluripotent stem cells (iPSCs). It is important to consider the proliferative capacity, differentiation potential, suitability in scalable bioprocessing systems, and their impact on the structure and sensory properties of muscle tissue. This analysis demonstrates that no single cell type can fully replicate the complex structure of native muscle tissue. Satellite cells are responsible for the formation of muscle fibers, fibroblasts provide support through the synthesis of the extracellular matrix, and adipocytes contribute to the flavor and juiciness of the final product. Pluripotent cells differentiate into all of cell lineages, but their use is associated with regulatory and ethical considerations. This work also addresses key aspects of bioprocess engineering, such as scalability, culture conditions, and the importance of 3D cell culture and cell co-cultures in restoring tissue structure. Furthermore, regulatory, ethical, and economic issues affecting the feasibility of implementing the technology for industrial production are considered. In summary, the data presented indicate that the development of cultured meat requires an integrated approach combining appropriate cell selection, process optimization, and compliance with regulatory and ethical requirements.

## 1 Introduction

**Figure 1:**
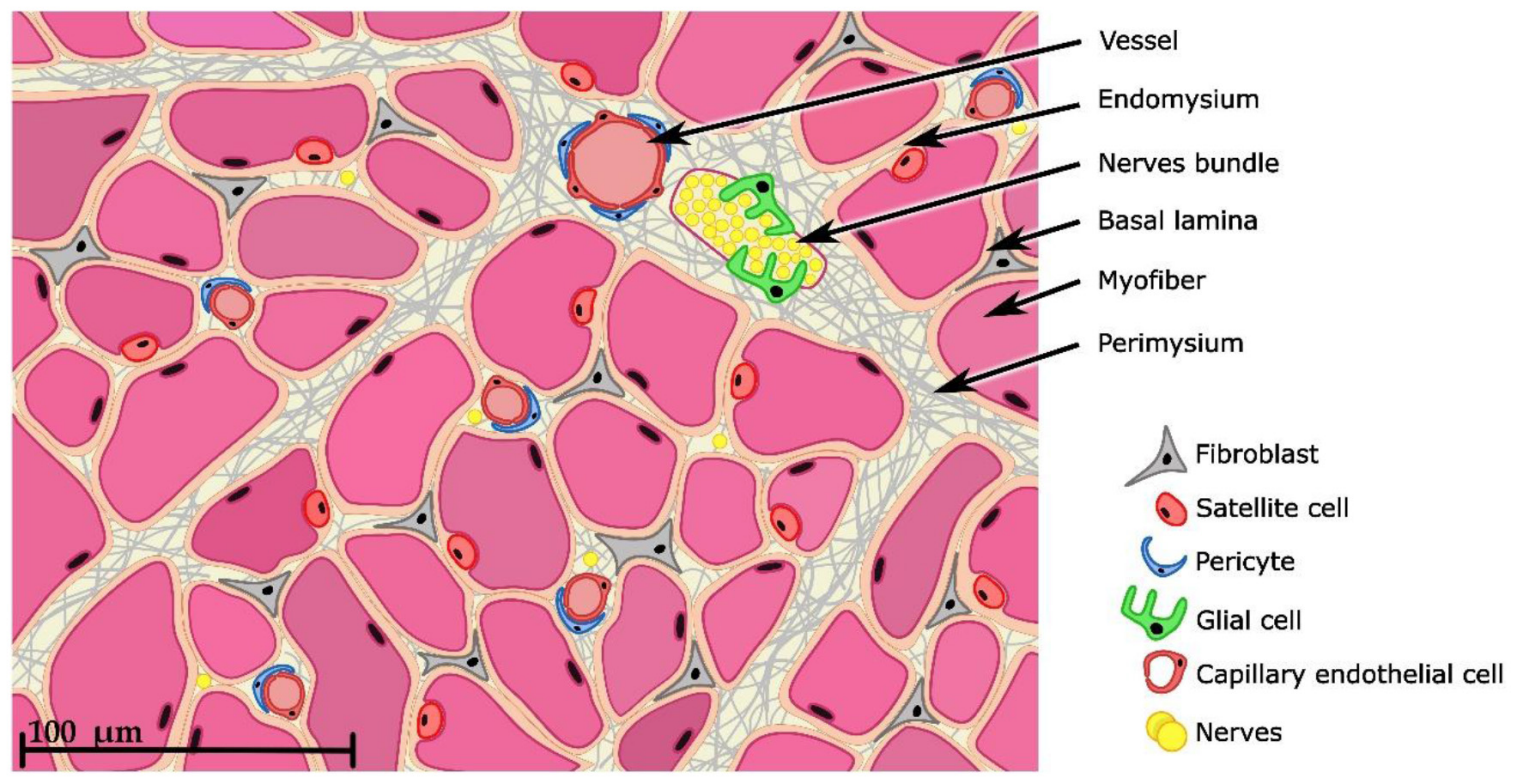
The cross-section of the mammalian anterior tibial muscle. Reproduced from (166) with Inkscape 1.2.2, licensed under GNU General Public License v3.

Meat consumption is projected to increase due to the growing human population (1). Meat production raises several controversial issues, including welfare concerns related to animal rearing conditions. As global affluence rises, more people wish to consume meat while concurrently considering the ethical implications of its production. Consequently, alternative strategies for meat production are being actively investigated. Cultured meat (lab-grown meat) has recently attracted considerable attention. However, *in vitro*–produced meat must closely resemble the structure of meat obtained from conventional livestock farming (2) (Figure 1). Bryant and Barnett (3) suggest public attitudes toward cultured meat are not entirely negative, although some skepticism persists. Flavor represents one of the most critical challenges in the meat industry (4). Accordingly, Lew et al., (5) aimed to generate cultivated pork fat for use in meat substitutes. Their results indicate that *in vitro* fat tissue is comparable to native porcine fat, highlighting its potential for food production. Mission Barns (San Francisco, California, US) cultivates pork fat using a patented bioreactor technology designed to enhance production efficiency. The cultivated fat is blended with plant proteins to produce items such as bacon and sausages. The company focuses on pork fat rather than whole cuts of meat because it is faster and more cost-effective to grow, while exerting a substantial impact on flavor and texture when combined with plant proteins. Their approach utilizes non-genetically-modified porcine cells grown in animal-component-free, serum-free media. Instead of conventional suspension bioreactors, Mission Barns employs a proprietary adherent bioreactor system that better mimics natural fat cell growth, reduces mechanical stress, and supports both cell expansion and scalable culture production (6).

Notably, the definition of “meat” in terms of cell-cultured products remains complex and subject to significant regulatory and cultural differences across the world. Meat is defined as muscle tissue composed of actin and myosin, of animal origin (7). CM can consist of various cell types, such as satellite, adipogenic, and supporting cells. However, the functional contribution of individual cell types to tissue structure, extracellular matrix (ECM), and sensory properties does not always align with current legal definitions of meat. As a result, some ambiguity exists regarding whether products composed solely of supporting cells, such as fibroblast-derived biomass, can be considered “meat” (8). In many jurisdictions, the regulatory framework is still being developed and evolving, and criteria may depend on characteristics such as: 1) cell origin, 2) degree of tissue organization, and 3) similarity to conventional meat. As a result, products consisting primarily of fibroblasts or ECM-producing cells may not be considered meat, despite their potential contribution to the texture and structure of the product (9).

In summary, cultured meat is a promising, albeit complex, technological and social innovation. Its progress will depend on the progress in the field of nuclear engineering, but also on the ability to adapt to the regulatory framework and to take into account the perception of consumers in different markets around the world.

## 2 The fibroblasts—a basic platform of unique cells

Among the non-myocytal cells, fibroblasts are the most numerous cells of the skeletal muscles, primarily responsible for synthetizing ECM structural proteins and remodeling its structure (10).

During growth and regeneration, i.e. during injury, fibroblasts proliferate, concentrate around injured sites, and therefore support regeneration and reconstruction (11). *In vitro* studies demonstrated that direct contact between fibroblasts and myogenic cells enhances the proliferation, differentiation, and fusion of the muscle cells (12). Fibroblasts also interact with satellite cells, the resident stem cells of skeletal muscle, promoting their differentiation and proliferation by secreting proper growth factors (13). Despite not being the target muscle tissue itself, fibroblasts have become a significant cell type in cultured meat research for several compelling reasons, such as simplicity in isolation, culturing, and proliferation (14). To ensure high proliferation of cells that will be used in meat culture, genetic modifications also could be applied (15). One includes suppression of cell cycle inhibitors, such as p21, p27, PTEN, p53 and cyclins (15). The main concern about genetic engineering is the impairment of cells' ability to differentiate. Indeed, some interventions can block differentiation and induce histone hyperacetylation in stem cells by impairing DNA methylation. Surprisingly, they do not affect the expression of typical embryonic stem cell markers, such as Oct4 (16). Fibroblasts can undergo numerous cell divisions before senescence, allowing for rapid biomass growth, which is crucial for scaling up cultured meat production (17). Yang et al., (155) described a cost-effective method for isolating fibroblasts derived from porcine skin tissues. With the use of physical friction and enzymatic digestion, about 9 times more cells were achieved compared to the standard explant method. Fibroblasts are characterized by a higher proliferation rate compared to muscle cells *in vitro*. Therefore, the ability to obtain 3D-printed fibroblast-cultured meat may provide an economical alternative. The use of 3D bioprinting and spheroid cell cultures on hydrogel bioinks creates opportunities to mimic natural conditions and produce porcine meat *in vitro* (165). Due to the secretory activity of fibroblasts, their addition to *in vitro* meat production affects the content of collagen-related amino acids (hydroxyproline, proline, and glycine) (155). Taking together, the ease of isolation and rapid proliferation make fibroblasts a promising model for meat cultivation.

### 2.1 Avian fibroblasts

For cultured meat purposes, specific fibroblasts isolated from chicken embryos were examined (18). Chicken embryo fibroblasts (CEFs) possess the main feature that is crucial in cultured meat research, which is that they are easy to source. In addition, they are more expansive, proliferative and less prone to senescence than mature fibroblasts (19). Furthermore, some research demonstrated spontaneous immortalization of CEFs, generating stable cell lines that grow well in serum-free media, which is a major step toward scalable and ethical production, also offering cost reductions as provided by the Believer Meats (Rehovot, Israel) company's empirical data (20, 21). The Food and Drug Administration (FDA) has approved the use of chicken fibroblast cells for cultured meat production. Eat Just Inc. has used fibroblasts cells, exactly commercially available DF-1 cell line (UMNSAH/DF-1, CRL-3586™, ATCC^®^), for the commercial production and sale of cultured meat in both Singapore and the United States (22). A recent study further confirmed the feasibility and consumer acceptance of cultured meat derived exclusively from native chicken fibroblast cells, marking a significant advancement (20). During the FDA pre-market consultations for food made with cultured cells (Cell Culture Consultation; abb. CCC), company notes (CCC000001) that this line is spontaneously immortalized, non-GMO, certified free of a range of avian pathogens, and was ordered from ATCC on February 22, 2018, then relabeled internally as “C1F” (23–25). However, three-dimensional constructs composed of fibroblasts cannot be labeled as a meat. Using fibroblasts as a filler or supplement may affect the properties of farmed meat, but it will not constitute it. Development of avian adipose tissue is also specific, due to the origin of triacylglycerol. In avian, it primarily stems from egg yolk (during embryonic development) and from circulating lipids in the blood (after hatching). In avian species, unlike in mammals, *de novo* lipogenesis occurs in the liver rather than in adipose tissue (26). It is possible to direct fibroblasts into adipocyte-like cells without direct genetic modification. Proper chemical or environmental induction, such as small molecule treating (STK287794 molecule), or injury healing process can mobilize fibroblast-to-adipose-like and adipose-to-fibroblast-like transition (27, 28). These methods influence gene expression and cell morphology, promoting PPARγ expression or shaping round adipose-like cells.

Cell expansion and proliferation are still the most desirable processes during biomass growth, which may occur under traditional 2D culturing, as demonstrated by Kim et al. (29). The researchers cultured CEFs on collagen-coated flasks, determining the doubling time at about 1.6 times faster than parallel cultured satellite cells. Even when compared with adipose-derived stem cells, muscle cells, and other stem cell types, they demonstrate a shorter doubling time makes CEFs a promising candidate for enhancing meat biomass production *in vitro* (30).

**Figure 2:**
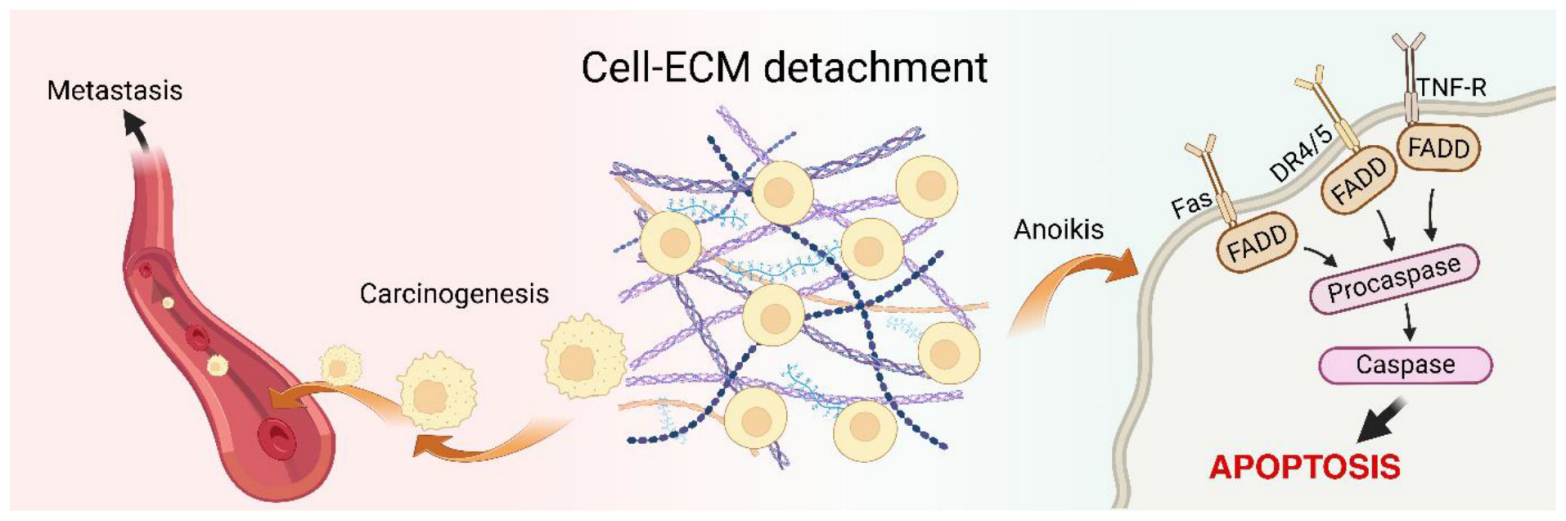
The role and way of action of anoikis, cell death specific to the detachment of a cell from the extracellular matrix.

Using 3D systems may additionally promote proliferation and forming three-dimensional cell constructs, as spheroids or organoids. As suggested by Ma et al., cells cultured on hydrogel scaffolds may more efficiently form myotube-like structures under overexpression of the transfected MyoD gene, compared to flat surface culturing (31). After coating on other 3D biomaterials, such a soy protein scaffold, CEFs may ultimately compose a cell-plant complex mimicking meat (20). Most cell types, cultured either two- or three dimensionally, are anchorage-dependent. They require attachment to a surface to avoid a programmed cell death process known as anoikis, shown at Figure 2 (32). Suspension cell culture systems are generally more efficient than adhesive systems for large-scale cultured meat production. Suspension cultures allow for higher cell densities, improved scalability, and more effective control of environmental parameters within bioreactors, thereby reducing labor and cost. In contrast, adherent cultures are limited by surface area requirements and are more suitable for applications requiring structured tissue formation. The sources show that fibroblasts can not only be adapted to suspension growth, but that such suspension-proficient fibroblast lines are explicitly positioned as a promising tool for scalable, industrial cultivated meat production. Pasitka et al. optimized fibroblasts for suspension by long-term adapting cells to serum-free, non-adherent conditions, gradually increasing the swirling of the suspension, ultimately yielding a stable, genetically unmodified fibroblast line that grows at density of 108 × 10^6^ cells per milliliter (20). Moreover, Pasitka el al. employed spontaneously immortalized CEFs without any genetic manipulation, which could help alleviate public concerns about GMOs and genetically modified food. Pan et al. also utilized long-term culture to adapt fibroblasts, the DF-1 line, for suspension culture reaching 2 × 10^6^ cells/mL, which does not reach the optimization level reported by Pasitka et al., but still shows a significantly enhanced efficiency to the adhesive model (33). A deeper transcriptomic analysis of DF-1 cells showed that the key for adaptation is suppressing non-essential metabolism and regulation of cell junctions in response to oxidative stress and cell detachment, regulating genes as MAPK, TEAD, YAP and FoxO1 (34).

Summarizing, chicken fibroblasts are easily accessible and exhibit rapid growth, which makes them the first target population commercialized by by companies such as EatJust Inc., Believer Meats, and Upside Foods. Nevertheless, CEFs are obtained from embryonic material (usually around the 10th-11th day of development). Although it does not involve the slaughter of adult animals, it is based on the collection of embryos, which may raise some ethical and regulatory concerns and affect consumer acceptance related to the source of the cells.

### 2.2 Fish fibroblasts

Fish fibroblasts demonstrate potential to transdifferentiate into various cell types essential for meat production, without genetic manipulations (35). Currently, research on the cultivation of fish meat is dominated by studies on muscle and fat cells, largely omitting the role of fibroblasts or fibroblast-like cells (36). While fibroblast-like cells are mentioned in research papers, where myoblasts undesirably gained a fibroblast-like morphology, gradually dominating and overgrowing the muscle cell population (37, 38). The first attempts to cultivate fish meat assumed a culture system in which myocytes and fibroblasts were co-cultured (39). In the fish meat structure, most fibroblasts are located in the muscle septa, where they synthetize ECM components, including collagen type I and V (40). Notably, fish fibroblasts do not synthetize collagen type III, one of the a major collagenic component in mammalian muscles, because they do not have an orthologous gene encoding for *col3a1* (41). Despite the muscular location of fibroblasts, fish skin and the fin are the most commonly used niches for isolation. Fish fin tissue, especially caudal, is rich in fibroblasts and has strong regenerative capabilities. The fibroblasts isolated from fin exhibit efficient growth and proliferation on 3D scaffolds, potentially enabling meat biomass growth (35). Fibroblasts isolated from fish skin show high proliferation, also on scaffolds (42). In summary, fish fibroblasts are not widely described in meat production but their localization in the intermuscular septa and three-dimensional expansion capabilities suggest their potential application as an element of the cultured meat biomass.

### 2.3 Porcine fibroblasts

Porcine fibroblasts have been isolated and studied for cultured meat production. Immortalized porcine fibroblasts were employed to generate meat aroma precursors by freezing and thawing, which can be incorporated into plant proteins, leading to the production of tissue, sensorially corresponding to pork meat (43). Growth factors, including the fibroblast growth factor (FGF-1), are required for proper and efficient cell growth in *in vitro* culture. Another way to improve the efficiency of meat culturing could be the use of recombinant porcine FGF-1 (rpFGF-1). An efficient method for expression and purification of rpFGF-1 could promote scaling cultivation. When applied to cultured porcine muscle stem cells (MuSCs), it significantly affected growth, improved mitochondrial function and proliferation of MuSCs, even under fetal bovine serum (FBS) deprivation (44). Modification of the GDF8 gene using the CRISPR/Cas9 system, resulting in a myostatin-deficient phenotype and altered regulation of muscle development in Debao pig fibroblasts and embryos, allows for increased efficiency in muscle production (45). Bovine embryonic fibroblasts, may be transdifferentiated into other cell types, also muscles (46). Bovine fetal fibroblasts reprogrammed to bovine induced pluripotent stem cells (iPSCs) are in use for myogenic differentiation. The cell line generated with episomal vectors (pMaster K and pCXB-EBNA1) was stable over multiple passages and expressed pluripotency markers (e.g., AP, SOX2, NANOG), followed by elevated levels of the myogenesis marker PAX3. The cells in culture resembled myotubes not only morphologically (elongated nature and multiple nuclei) but also had similar gene expression profile (47). Immortalized bovine embryonic fibroblasts transformed with MyoD and PPARγ2 were used for hydrogel scaffold-based 3D printing, followed by myogenesis and adipogenesis after incubation with the appropriate differentiation medium (48). By hydrogel 3D printing, it is possible to obtain a heat-treatable product that retains its original shape. The use of textured soy protein as a scaffold for expansion of bovine cells to obtain meat *in vitro* was also studied (49). The scaffolds provide the basis for obtaining a three-dimensional structure, creating the possibility to populate various cells that refer to the natural texture of meat.

A major limitation of *in vitro* cultivation is the significant cost, which includes expensive growth medium ingredients. The fibroblasts, as stated above, are relatively easy to immortalize, either spontaneously or through genetic modification, and they exhibit robust proliferation that make them highly amenable to large-scale cultivation *in vitro*. Their stability and adaptability under serum-free conditions make them attractive for cost-effective, high-density cell culture systems. The use of FBS is associated with high costs and also presents ethical issues. There are attempts to reduce the use of FBS or to use other alternative ingredients. Bovine fibroblasts cultured in *Chlorella vulgaris* algae extract, represents two growth factors (FGF-2, TGF-β1), and insulin growth and enhanced viability. However, the proliferation was still lower when compared to standard media with FBS (50). The use of FBS in cell culture is associated with significant limitations, which include high cost, compositional variability, and ethical concerns. Given these limitations, efforts are underway in the context of meat production to develop FBS-free or chemically modified media. Defined media would enable the standardization and scaling of the process. Recombinant growth factors are used, such as fibroblast growth factor (FGF) or insulin-like growth factor (IGF) are essential components of most serum-free media compositions (51). Additionally, alternative sources of nutrients derived maybe from plant extracts or algae (8).

Secondly, there remains little standardization for isolation and characterization between species, and the variable content of fibroblasts in muscle tissue contributes to the fact that during isolation, unfiltered adipose tissue may contain different proportions of cells with different functional properties, e.g. Fibro-Adipogenic Progenitors (FAPs), resident interstitial fibroblasts, and perivascular fibroblasts, which may affect the ECM content (52). It is also worth noting that fibroblasts under specific culture conditions can differentiate into myofibroblasts, which are characterized by *de novo* expression of smooth muscle α-actin (α-SMA) and increased contractility (53).

Work should continue on developing species-specific markers and standardizing the isolation protocol. It is also worth optimizing the conditions for fibroblast co-culture with MuSC to obtain the native structure of muscle tissue.

## 3 Satellite cells: advantages and disadvantages

Muscle satellite cells (MuSC) are mononucleated myogenic muscle stem cells of mesenchymal origin, located between the basement membrane and the sarcolemma of terminally-differentiated muscle fibers (54), they were first described by Alexander Mauro (55). Under normal conditions, these cells remain in a quiescent state in mature skeletal muscle. They act as a reserve population of progenitor cells capable of proliferating in response to injury or other stimuli, giving rise to proliferating progenitors that drive muscle regeneration and repair, while preserving the stem cell pool through self-renewal (56). Their ability to proliferate and differentiate into myoblasts, which subsequently fuse to form myotubes and adult muscle fibers, renders them essential for muscle growth and regeneration *in vivo* (57). In the context of cultured meat production, MuSC are indispensable for the generation of skeletal muscle tissue, the primary component of conventional meat. Currently, MuSC are cultured together with adipogenic cells (e.g., preadipocytes, adipocytes) or supporting stromal cells, such as fibroblasts or Fibro-Adipogenic Progenitor cells (FAP cells), which enables the reconstruction of the native muscle tissue structure, as it leads to: formation of aligned muscle fibers, incorporation of intramuscular fat. As a result, it shapes the organoleptic characteristics of conventional meat such as: juiciness, and tenderness (49).

MuSC offer several advantages for cultured meat production. Upon activation from quiescence, these resident skeletal muscle stem cells can undergo multiple population doublings (PDs) while retaining myogenic competence, enabling expansion to the cell numbers required for industrial-scale production. In contrast to induced pluripotent stem cells (iPSCs), which are generated by somatic cell reprogramming and require careful evaluation of genomic stability and tumorigenic potential, satellite cells are myogenic lineage-restricted and can be isolated without pluripotency-inducing reprogramming. However, this proliferative capacity is finite: during prolonged *in vitro* expansion, satellite cells undergo telomere attrition, enter replicative senescence, and progressively lose self-renewal and myogenic differentiation capacity (58). In living organisms, the number of MuSC per myofiber decreases with age, reveals that muscle samples from aged humans accumulate senescence markers (p16, p21, SA-β-gal) and exhibit cell-cycle arrest, demonstrating the presence of senescent MuSC in aging human muscle *in vivo* (32, 59). Numerous repeating injuries, and especially collagen IV deficiency, can also lead to depletion of the satellite cell pool (60). While a healthy environment can partially restore their function, they cannot divide indefinitely like immortalized or cancer cells (61). Thus, satellite cells are long-lived and highly regenerative, but not truly immortal. Immortalization through genetic modifications could improve viability. Immortalization through genetic modifications could improve their viability. Several methods of immortalization have been described; for instance, telomerase reverse transcriptase (TERT) overexpression is the most widely used (15, 62). Transfection of bovine satellite cells with Sleeping Beauty plasmid containing bovine TERT and CDK4 is one example (63). Two other methods can also enable immortalization, i.e., SV40 overexpression or inhibition of the expression of the selected genes that selected cell-cycle regulatory genes (15). Stout et al., (64) investigated whether immortalized bovine satellite cells with FGF-2, RasG12V, or both FGF-2/RasG12V overexpression could be beneficial for cell-based meat production. This myogenic differentiation capacity of satellite cells is central to replicating the structural and functional properties of meat, including its fibrous texture, protein composition, and organoleptic qualities. Moreover, satellite cells are genetically stable across early passages (65), as they maintain a consistent genomic and epigenetic profile that minimizes variability in the final product.

Furthermore, MuSC can be isolated from various livestock species including, chicken, pig, cow (beef) and sheep, thereby enabling the production of diverse meat types tailored to consumer preferences and dietary habits across the world. Chicken satellite cells are relatively easy to isolate and culture, under defined conditions with moderate efficiency. Pig satellite cells exhibit robust proliferative and differentiation capacities, making them ideal for replicating the characteristics of conventional pork (66). Bovine (beef) satellite cells exhibit robust proliferation and good differentiation potential, supporting large-scale production of beef-like tissue (67). In sheep, several studies have suggested satisfactory differentiation potential and proliferation rates comparable to bovine or porcine cells (68), a lack of direct comparative studies between ovine, bovine and porcine MuSC. Fish satellite cells, while isolatable from salmon, tilapia and trout, require lower culture temperatures and tailored growth-factor formulations (69–71) and their population-doubling time of approximately 28 h necessitates species-specific optimization (72, 73). Fish satellite cells may require surfaces or scaffolds made of ECM proteins such as elastin, collagen, fibronectin, and laminin to adhere and optimization of these substrates is critical for efficient fish cell culture (74, 75).

The use of MuSC in meat production is fraught with several challenges. One of the primary limitations is the technical difficulty in isolating and purifying MuSC from muscle biopsies (76), especially for species that have not been extensively studied. In addition, the standardization of isolation protocols is species-specific and laborious. Moreover, MuSC are exquisitely sensitive to their *in vitro* microenvironment (77). Therefore, the culture conditions, including oxygen tension, substrate stiffness, medium composition and temperature, need to be meticulously optimized for each species to sustain proliferation without premature differentiation or loss of myogenic potential. For example, fish satellite cells often require significantly lower culture temperatures and modified media formulations compared with mammalian cells. The intrinsically different culture conditions of fish compared to mammalian cells can create logistical barriers to large-scale production and complicate bioprocess engineering efforts. Another problem is the cost of satellite cell cultured meat production (78). High-grade serum-free media, which are supplemented with essential growth factors (GFs), such as insulin-like growth factor-1 (IGF-1), epidermal growth factor (EGF), and hepatocyte growth factor (HGF), are prohibitively expensive, although the development of cost-effective alternatives is an active area of research. Another significant obstacle is scalability (79). Although small-scale expansion of satellite cells has been demonstrated in laboratory settings, transitioning to industrial-scale bioreactors involves numerous technical hurdles, including maintaining homogeneous cell populations, preventing senescence, and ensuring efficient nutrient delivery within cultures. MSCs, just like fibroblasts are adherent cells that require a surface for attachment. 81 showed that when MuSC are cultured in suspension aggregates they tend to enter a quiescent, non-proliferative state rather than expanding efficiently (80). Currently, one of the most approach for scaling is the use of microcarriers in stirred bioreactors, i.e., treating satellite cells as anchorage-dependent and providing surfaces in a “pseudo-suspension” rather than true single-cell suspension (81). This method, although similar to suspension culture, still limits growth to the available surface area, but allows improved control of nutrient and oxygen delivery compared with traditional adhesion culture.

MuSC have a finite replicative capacity *in vitro* due to intrinsic aging mechanisms, including oxidative stress, telomere attrition, DNA damage responses (DDR), and activation of stress response pathways such as the p16INK4a/pRb and p53/p21CIP1 pathways (82). Furthermore, each source of MuSC presents unique challenges. Chicken MuSC cultures, for example, rely on FGF-2, which can significantly increase the complexity and cost of production. Fish MuSC often require species-specific optimization of temperature, GFs (e.g., FGF-2 and IGF-1), pH, osmolality, serum type and concentration.

MuSC represent a biologically versatile platform for cultured meat production. These cells offer several advantages, including high genomic and epigenetic stability, robust proliferative potential, measured in PDs, as well as adaptability across multiple livestock species. Such attributes make them promising candidates for replicating the physiological, nutritional and sensory characteristics of conventional meat. During differentiation, MuSC exit the cell cycle, fuse to form multinucleated myotubes, and synthesize contractile proteins, such as actin and myosin, which are essential for the texture and functionality of meat products. However, generating immortal and scalable cell lines is challenging because these cells tend to terminally differentiate, and their practical utility is further constrained by challenges such as difficult isolation and purification, lack of standardized culture protocols, limited scalability, and high production costs. Overcoming these hurdles will require interdisciplinary efforts, ranging from improvements in isolation and cell-sorting technologies to the development of cost-effective media, scalable bioreactor systems, and a deeper understanding of species-specific muscle biology.

## 4 Embryonic stem cells—are they a holy grail of cultured meat?

The production of cultured meat is becoming one of the most noteworthy potential solutions for sustainable animal farming, namely eliminating the need for slaughter and limiting greenhouse gas emissions. An important aspect of the production of this type of meat is the selection of the right type of stem cells that will effectively proliferate and differentiate into muscle cells, e.g. embryonic stem cells (ESCs). As a result, this chapter presents the advantages and future challenges associated with the use of ESCs from chickens, pigs, beef, sheep, and fish for the production of cultured meat.

ESCs are pluripotent cells derived from blastocytes, which are characterized by the ability to undergo unlimited self-renewal and to differentiate into any type of adult cell. Furthermore, ESCs can also be repeatedly modified and then used as nucleus donors in the cloning process (NT), which results in rapid improvement of breeding characteristics. The development of ESC lines obtained from farm animals was limited for many years due to difficulties in maintaining their pluripotency *in vitro*, but protocols have now been developed that allow their stable cultivation from species such as pigs, chickens, cattle, fish, or sheep. However, they convert to the germ line with low efficiency, e.g. in pigs or chickens, which is the reason that research efforts have been somewhat limited (66).

In recent years, there has been growing interest in the use of bovine stem cells, particularly blastocyst-derived pluripotent cells, in the context of novel biotechnology applications such as cultured meat and cell bioreactors for the production of animal proteins. Bovine embryonic stem cells show potential for long-term culture and differentiation into various somatic cell types, including myoblasts and adipocytes, which are major components of meat (152). The main challenge is the inability to obtain stable pluripotent cell lines that meet all functional criteria, including long-term self-renewal, expression of pluripotency markers, ability to differentiate into cells of all three germ layers, and potential to integrate into the germline of host organisms (164). Previous attempts to obtain bovine ESCs have mostly been based on isolating cells from early blastocyst stages and culturing them in the presence of growth factors such as leukemia inhibitory factor (LIF) and basic fibroblast growth factor (bFGF). The resulting cell lines showed ESC-like properties, but often lost their ability to self-renew after several passages and their differentiation potential remained limited (154, 162). There are promising reports demonstrating the possibility of reprogramming these cells to maintain their proliferative and differentiation capacity *in vitro*. Recently, iPSCs are being increasingly studied. iPSCs are somatic cells that have been reprogrammed to a pluripotent state and have properties similar to embryonic stem cells, such as: self-renewal and the ability to differentiate into cells of all three germ layers. Moreover, iPSCs can be easily obtainable from adult tissues avoiding ethical concerns associated with embryonic stem cells also can be expanded and genetically optimized for improved growth, differentiation efficiency. It is also worth noting that ESCs have higher developmental potential compared to iPSCs, namely in livestock species (157) ESCs sourced from blastocysts and have a natural ability to pluripotency and a predisposition to differentiate into adipose lineages and muscle without reprogramming and iPSCs require reprogramming protocols which enables them differentiation efficiency (151). ESCs remain a valuable platform, particularly in industrial purposes where high differentiation capacity is critical.

ESCs derived from blastocysts naturally have a higher developmental potential, making them ideal for differentiation into muscle and fat cells while maintaining safety (156). The breakthrough came with research on bovine embryonic stem cells (bESCs), in which special media with Activin A, FGF-2, Wnt/Tankyrase pathway inhibitors (e.g., IWR1, XAV-939) were used, which enabled the preservation of pluripotency. It is also worth emphasizing that the differentiation protocol has been successfully used in pigs and other species (158).

Pigs represent an important source of embryonic stem cells. First porcine embryonic cell line was produced by Piedrahita et al. (83). Using procedures already known from the isolation of embryonic cells from other species, mouse ESCs, he successfully isolated and established a culture of pig cells (83). In 2019, Choi et al. obtain stable lines of embryonic cells of porcine origin, namely by culturing blastocysts in special media with FGF-2, Activin A, CHIR99021 (GSK3 inhibitor), and Wnt pathway inhibitors. The cells were characterized by the expression of pluripotency markers (NANOG, OCT4, SOX2) (84). Porcine pre-gastrulation epiblast stem cells were created and proliferated for more than 240 passages. Kinoshita et al. (85), described ESCs from cattle, also achieved this state in pigs by using the same culture conditions. Bilaminar discs from day 11 embryos were dissected, and the epiblasts were plated to derive pESCs. pESCs exhibit pluripotency features and genome stability (85). Another aspect is the effective differentiation of muscle cells to obtain cultured meat. A cultured meat production process may have stages such as: proliferation, differentiation into muscle cells and meat structuring. Zhu et al. (86) isolated 5.3 × 10^4^ muscle stem cells. However, during long-term culture, these cells lose their stemness, namely, there is a decrease in the expression of stem cell markers, e.g. Paired Box 7 (PAX7) and mature muscle cells, such as Myosin-heavy-chain (MyHC). Differentiation is associated with increasing oxidative stress, which reduces the ability of these cells to transform into muscle structures. Therefore, it is important to use factors supporting proliferation and differentiation that counteract these processes. An example is supplementation of the medium with L-ascorbic acid 2-phosphate (Asc-2P) at a concentration of 100 μm, which improves the condition of the cells. Asc-2P reduces oxidative stress, which enables these cells to proliferate and then differentiate (H. 87).

**Figure 3:**
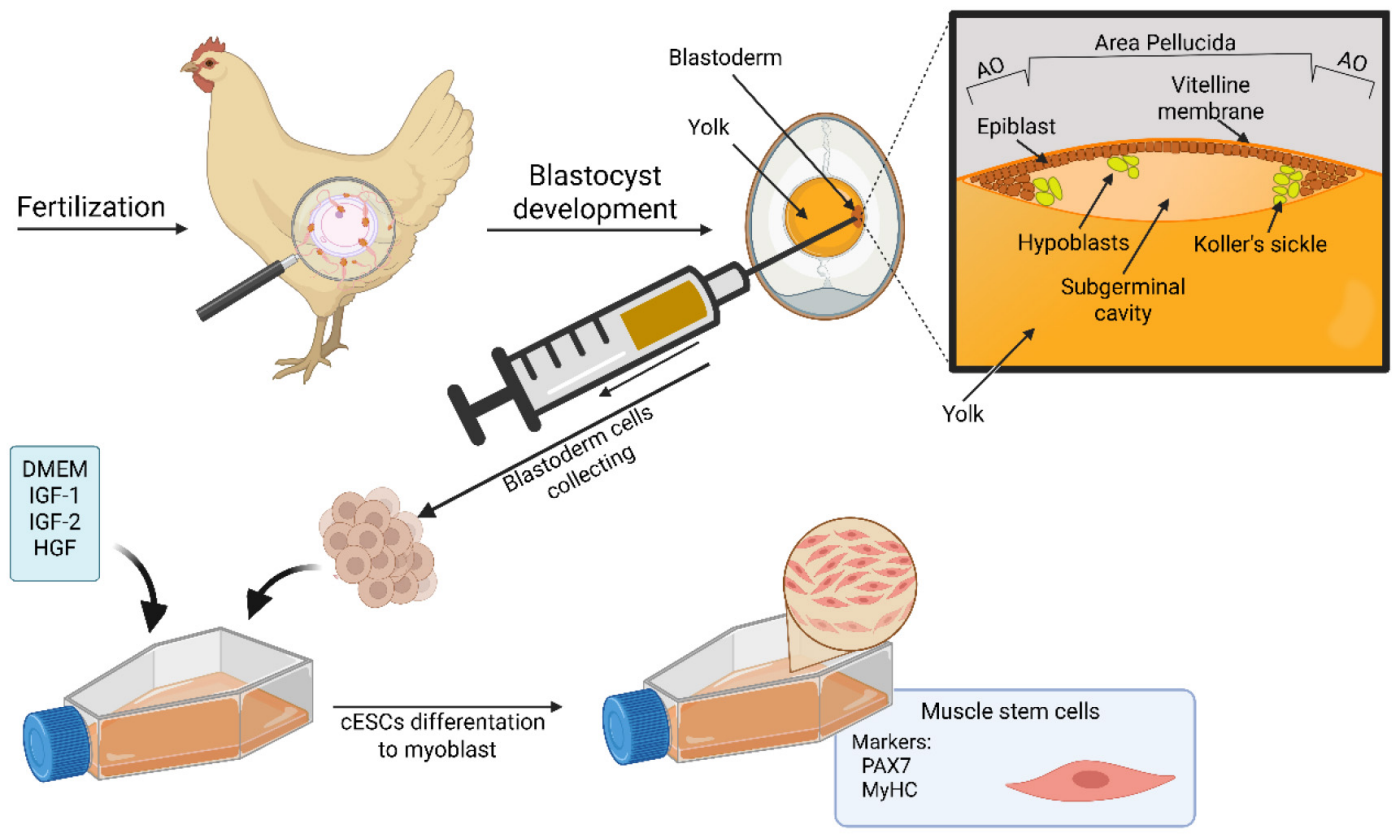
Graphical representation of sourcing, culturing and characterization of chicken embryonic stem cells. Blastoderm structure at X stage of chicken development is also represented. AO, area opaca.

Chicken-derived ESCs (cESCs) play an important role in many areas of research, including meat production (Figure 3). Companies such as LabFarm or other startups are already using cESCs as a production platform for cultured poultry meat. cESCs represent one of the first platforms for serum-free vaccine manufacturing, ESCs can proliferate under chemically defined (non-GMO) conditions. Nevertheless, the main challenge for cultured meat applications is controlled differentiation into muscle and fat lineages, which is why research in this area is important (163). However, a widely used culture system for these cells has not yet been developed to enable their widespread use. One example of a culture system for cESCs that maintains pluripotency is the RLSF system. This system uses reagents such as LIF, SCF (stem cell factor), FGF-2, as well as the use of PD0325901 and SB431542 inhibitors and LIF factor (R2i+LIF system) to maintain the pluripotency of cESCs (87). cESCs also show the potential to differentiate into muscle cells using an appropriate medium such as: DMEM (Dulbecco's Modified Eagle Medium), F-10 Nutrient Mixture, IMDM (Iscove's Modified Dulbecco's Medium), as well as when appropriate molecular factors are added to promote muscle cell differentiation. The first of these is IGF-1 / IGF-2 (insulin-like growth factors), which promotes muscle differentiation and hypertrophy of muscle cells, as well as myoblast fusion. HGF (hepatocyte growth factor) promotes myoblast migration and proliferation (88).

In contrast to cattle or pigs, sheep are less often considered as a target species for meat production. The generation and maintenance of stable sheep embryonic stem cell (ESC) lines has long been a major challenge. Unlike well-characterized rodent and non-human primate ESCs, sheep ESCs did not exhibit the characteristics of full pluripotency or the ability to be cultured for long periods *in vitro*. Attempts to maintain the pluripotency of sheep cells under conditions adapted from mouse and human systems have been unsuccessful which may have revealed species-specific biology (148). To date, ESC-like cell lines have been successfully derived from early sheep blastocysts. Zhao et al. (161) described the successful derivation of sheep embryonic stem-like cells (ESC-like cells) cultured under semi-defined medium conditions without the use of feeder cells. These cells expressed pluripotency-specific markers such as octamer-binding transcription factor 4 (Oct4), homeobox protein (NANOG), SRY-box transcription factor 2 (Sox2), and stage-specific embryonic antigen-1 (SSEA-1), and retained the ability to form an embryoid body and differentiate into cells derived from three embryonic layers. A breakthrough in this field came from the study by Vilarino et al. (150), where the authors successfully derived stable ovine ESC lines using a chemically defined culture system containing FGF-2 and a tankyrase/Wnt inhibitor (IWR1). The resulting cells were characterized by classical dome-shaped colony morphology, stable expression of pluripotency markers and a euploid karyotype over more than 40 passages. The presence of FGF-2 and IWR1 was shown to be critical for maintaining the undifferentiated state of ESCs derived *de novo* from sheep blastocysts. These results represent a significant step forward not only in understanding the mechanisms that regulate pluripotency in farm animals, but also in potential applications of sheep ESCs in biotechnology, biomedicine and agriculture, including animal meat production systems, disease models and advanced embryo engineering techniques.

In difference to the well-studied mechanisms of pluripotency in mammals or chickens, knowledge of fish ESC-like cells is an increasingly important area of ESC research, offering a number of unique advantages that are also relevant to biotechnology applications such as *in vitro* cultured meat production. The striped fish (Danio rerio) is considered an ideal vertebrate model organism in developmental biology and genetics. In the early 1990s, it was possible to isolate cells with pluripotent properties from the blastula and gastrula of this species that differentiate into ectodermal, endodermal and mesodermal lineages (153). In recent years, the development of genome editing techniques, such as CRISPR/Cas9, has opened up new possibilities for using fish ESCs to generate transgenic lines and human genetic disease models. In addition, CRISPR/Cas9 makes it possible to modify cell lines used for meat production because the technique may lead to improvements of genetic stability, growth rate and sensory properties of cultivated meat (159, 160). Important advantages of fish ESCs are their fast cell cycle, high proliferation and the ability to derive them from large numbers of embryos. These properties make them a promising source for the generation of muscle and fat cell lines, which is crucial for the production of cultured fish flesh (fish cells) (89). There have also been studies in other farmed fish species, such as Nile tilapia (Oreochromis niloticus), where it has been possible to derive stable ESC-like cell lines that exhibit pluripotency and the ability to form chimeras (147). Similar attempts have been made in other species, but results to date have been limited. In 2017, Fan et al., derived ESC-like lines (TES1-3) from Nile tilapia blastomeres. TES1 cells showed long-term growth without a feeder, expression of pluripotency genes (pou5f3, sox2, myc, klf4) and the ability to form embryoid bodies. In the long term, fish ESCs may serve not only as a source of biomass for human consumption, but also as a platform for the creation of personalized protein products with optimized nutritional composition. Furthermore, their proven use in large-scale vaccine production and immortal nature, as well as their ability to differentiate into different cell types without genetic modification, underscore their potential for scalable and versatile applications (89, 147).

Although various cell culture strategies are widely used in meat production, their suitability varies depending on several factors. First and foremost, these include scalability and production costs, and the ability of cells to differentiate. Although 2D adherent systems are well characterized and easy to maintain, they unfortunately have limited scalability and do not reflect the physiological tissue environment (8).

An alternative approach involves systems based on microcarriers, which not only increase the cell adhesion surface area but also enable cell culture in bioreactors. Unfortunately, this approach entails additional costs and requires the separation of the final product (90, 91).

Suspension cultures exhibit high scalability and are quite widely used in industrial biotechnology. Their main advantage is the ability to conduct processes in large bioreactors, along with greater environmental control and automation (Table 1). Unfortunately, many types of eukaryotic cells, especially mammalian cells, exhibit various degrees of adhesion to the substrate (92).

**Table 1:** Comparison of cell culture systems.

Cell culture system	Advantages	Limitations	Scalability	References
2D adherent culture	Simple to operate and enables precise control of culture conditions	Lack of 3D structure and low culture efficiency	Limited scalability	(8)
Microcarriers	Extensive surface area; suitable for bioreactors	The high costs and the need to separate cells from their carriers	Moderate scalability	(90, 91)
Suspension cell culture	Easy to scale; industrially applied	Limited differentation potential of adherent cells	High scalability	(92)
Scaffolds	Enables tissue structure reconstruction	Expensive to scale	Moderate scalability	(93)

This type of adhesion influences the regulation of their biological functions, proliferation, as well as survival and differentiation. This is closely linked to the activation of signaling pathways through contact with the ECM. These pathways influence gene expression. Under suspension culture conditions, the absence of appropriate adhesion signals can lead to cellular dysfunction, such as apoptosis or growth inhibition. Consequently, the use of this method has certain limitations for cells that require contact with a substrate. Microcarriers allows adherent cells to grow simultaneously in suspension while maintaining contact with the culture surface (93). Scaffold-based systems allow for the recreation of muscle tissue structure. Unfortunately, their large-scale application remains a challenge, which is primarily due to the high cost of materials and the need for strict control of the production process (94).

These insights demonstrate that despite their advantages, ESCs and iPSCs still require research into technical and regulatory aspects. Standardization of differentiation protocols, high production costs, concerns regarding genetic stability and safety, namely the use of viral vectors or genetic reprogramming techniques. Another problem is the elimination of animal-derived compounds in breeding systems, e.g., animal-derived substrates or feeder layers (e.g., mouse embryonic fibroblasts, MEF), as their elimination while maintaining the pluripotency of cells and their proper functioning is technically difficult and may affect the reproducibility and scalability of processes.

## 5 Mesenchymal stem cells (MSCs)

MSCs are multipotent progenitor cells isolated from various tissues, including adipose tissue, placenta, bone marrow, and skeletal muscle. In 2006, the International Society for Cell Therapy (ISCT) established a set of minimal characteristics to define MSCs. They are characterized by the expression of surface markers CD73, CD90, and CD105, as well as the lack of hematopoietic markers, fibroblast-like morphology, and the capacity to differentiate into adipocytes, osteocytes, chondrocytes, myocytes. However, it should be emphasized that these criteria were established for human cells, and MSC marker expression varies significantly between species, which necessitates careful evaluation when characterizing MSCs derived from other organisms such as poultry, fish, or cattle (95).

MSCs isolated from bovine adipose tissue exhibit expression of CD29, CD44, CD73, CD90, and CD105. Importantly, these cells can differentiate into adipocytes in both 2D and 3D cultures, maintaining a mature adipocyte phenotype for at least 2 weeks, and they also demonstrate myogenic potential. Notably, these cells are characterized by phenotypic stability and a high proliferation rate, making them a promising cell source for cultured meat (CM) (96). In pigs, MSC characterization is less standardized. Adipose-derived MSCs express CD29 and CD44 and are capable of differentiating into both myocytes and adipocytes; however, there is a lack of comprehensive data enabling cross-species comparisons. Currently, the FaTTy cell line an immortalized MSC line that retains strong adipogenic differentiation capacity over extended passages. Importantly, it does not exhibit tumorigenic properties and represents a promising source for large-scale production (97). MSCs from poultry, namely from embryos, express pluripotency related genes such as: NANOG, OCT4, SOX3, as a result of which they differentiate into adipose, cartilaginous and bone tissue. The cells exhibit a fairly rapid proliferation rate and phenotypic stability over several passages (98). Research is currently being conducted on the use of MSCs from fish, resulting in immortalized cell lines that differentiate into muscle and fat cells. Although there are no standardized protocols for isolation, markers for individual species are not available (99).

The second aspect may be the organoleptic properties of cultured meat derived from MSCs. MSCs have the ability to differentiate into adipocytes and myocytes, which allows for obtaining two essential components of meat from a single cellular source (100). Adipose tissue obtained from differentiated MSCs has a lipid profile similar to *in vivo* adipose. In cattle, monounsaturated fatty acids, such as oleic acid and saturated fatty acids such as palmitic and stearic acid dominate, which is similar to the composition of natural body fat. The FaTTy line has a higher ratio of monounsaturated to saturated acids than natural tissue.

In conclusion, the use of MSCs in the production of cultured meat still requires further research. The key stages will be detailed characterization of MSCs for different species and standardization of isolation and differentiation processes. However, MSCs are one of the promising sources of cells for cultured meat, due to their availability and multipotency, which leads to the regeneration all tissues found in the meat.

## 6 Adipose-derived stem cell (ADSCs)

ADSCs are mesenchymal stem cells and express surface markers such as CD29, CD34, CD73, CD90, and CD105, but do not express hematopoietic markers. ADSCs isolated from pigs express CD29, CD90, CD44, CD140b, and CD105, while those from cattle express surface markers such as CD73, CD90, and CD105, and lack CD56 expression, which allows them to be distinguished from satellite cells. On the other hand, ADSCs from chickens express CD71 and CD73 similarly to the above (101).

In the process of differentiating ADSCs into adipocytes, so-called adipogenic cocktails are used, including dexamethasone, IBMX (3-isobutyl-1-methoxyxanthine), rosiglitazone, indomethacin, and insulin. Dexamethasone is a synthetic glucocorticoid, IBMX increases cAMP levels and activates protein kinase A, rosiglitazone directly activates PPARγ, indomethacin inhibits anti-adipogenic prostaglandins, while insulin supports glucose uptake and lipid synthesis. As a result, the chemical compounds in the adipogenic cocktail activate the peroxisome proliferator-activated receptor (PPARγ), which regulates adipogenesis. Assessment of ADSCs differentiation into adipocytes most often involves lipid staining (Oil Red) also analysis of gene expression, such as PPARγ, and fatty acid profiles (102).

Interspecies differences in ADSCs are crucial for fat production in cultured meat, as they influence the ability to differentiate into adipocytes and the lipid composition of the final product. ADSC culture conditions are relatively uniform across species, although differentiation protocols are not standardized. ADSCs isolated from pigs and cattle maintain proliferation capacity and adipogenic potential until approximately passage 10, which differentiates them from satellite cells, which lose these properties more quickly. ADSCs from poultry can retain their differentiation capacity up to passage 37, while expressing PPARγ and accumulating lipids (103). Furthermore, immortalized porcine preadipocyte lines have been created that have the ability to proliferate and differentiate for over 40 passages. It is also worth emphasizing that ongoing work on optimizing ADSC culture allows for reduced FBS serum consumption through the use of growth factors such as Platelet-Derived Growth Factor BB (PDGF-BB), basic Fibroblast Growth Factor (bFGF), Epidermal Growth Factor (EGF), and Insulin-like Growth Factor 1 (IGF-1) (104).

Taken together, ADSCs are a key element in the production of fat for cultured meat, but the isolation protocol for these cells requires standardization and thorough cross-species characterization. Secondly, another aspect requiring further investigation is the co-culture of MuSC and ADSCs, as they have different differentiation conditions.

## 7 Dedifferentiated fat cells (DFAT)

DFAT cells are formed from mature adipocytes through a process of dedifferentiation. As a result, mature adipocytes in the ceiling culture change their morphology into fibroblast-like cells capable of proliferation, which is accompanied by a decrease in the expression of adipogenic markers such as adiponectin and peroxisome proliferator-activated receptor gamma (PPARγ). DFATs are multipotent, and as a result, they can differentiate into adipocytes, osteocytes, chondrocytes, and myocytes. Additionally, the expression of surface markers is similar to MSCs (105).

DFAT bovine cells are obtained from adipocytes isolated from subcutaneous or intramuscular adipose tissue. The dedifferentiation process takes 4 to 7 days, the doubling time of the population is approximately 20–22 h, and they also express genes characteristic of adipocytes. Although the data on the efficiency of adipogenesis in the later stages of cell culture are limited, there is no direct comparison to MSCs or ADSCs (106). DFAT from pigs have better adipogenic properties than those from cattle, i.e., they retain the ability to differentiate into adipocytes while maintaining PPARγ expression and intensive lipid accumulation even after 37 passages, and the adipose tissue obtained from pig DFAT has a fatty acid profile similar to that of natural pig tissue (107).

Broadly, despite significant results in pig models, DFAT cells are characterized by a complex process of their acquisition, unlike MSCs or ADSCs. Another difficulty may be the similarity of DFAT surface markers to MSCs and ADSCs, which makes their unequivocal identification difficult, as well as the lack of interspecies characteristics. Currently, DFAT cells of porcine and murine origin are most often described, while data for poultry and fish are practically non-existent, which makes it difficult to reliably estimate the usefulness of DFAT cells for cultured meat production.

## 8 Comparative analysis of cell types for cultured meat

### 8.1 Functional roles of cell types in muscle tissue

The production of cultured meat is not only based on the multiplication of individual cell types, but also on the reconstruction of the native structure of muscle tissue. Skeletal muscle is not only made up of myogenic cells, but is an integrated biological structure consisting of many cooperating cell populations, such as: MuSC, fibroblasts, adipocytes (93). MuSC participate in the construction of muscle fibers by differentiation and proliferation into myoblasts, while fibroblasts act as supporting cells by synthesizing ECM. Adipocytes accumulate lipids, which are necessary for organoleptic properties, such as taste and juiciness (108).

A single type of cell cannot replicate the native structure and function of conventional meat. The production of cultured meat must therefore be based on the interaction of different cell populations, as suggested in Table 2.

**Table 2:** Comparative analysis of cell types used in cultured meat production.

Criterion	Muscle satellite cells (MuSC)	Fibroblasts	Adipocytes/ MSC-derived adipocytes	ESCs/iPSCs (pluripotent cells)	References
Biological role	Formation of muscle fibers	ECM synthesis and structural support	Lipid storage, flavor contribution	Source of all cell types	(93, 108, 110, 113)
Proliferation capacity	Limited, subject to senescence	Very high	Moderate	Unlimited	(89, 109, 112)
Differentiation potential	Myogenic	Limited	Adipogenic	Pluripotent	(110–113)
Sensory relevance	Structure, texture, elasticity	Tissue stiffness (collagen)	Flavor, juiciness	Indirect (by differentiation)	(122–124)
Scalability	Limited (requires microcarriers)	Very high	Moderate	High	(117–119, 149)
Culture requirements	Adhesion-dependent, demanding	Low requirements	Differentiation-dependent	Very high (growth factors, control)	(89, 117, 119)
Regulatory end ethical aspects	High acceptance	High acceptance	High acceptance	Controversial	(124, 127, 128)
Technological risks	Senescence, loss of stemness	Low	Lipid variability	Heterogeneity, tumorigenicity	(111, 112, 123, 127)
Application in production	Muscle tissue formation	Biomass production, ECM support	Sensory quality improvement	Future platform	(149)

### 8.2 Proliferative capacity and biomass generation

The ability to maintain proliferation is a key factor determining the efficiency of livestock meat production. Fibroblasts have the highest proliferation potential. Moreover, they can be easily immortalized and grow well in suspension systems, which makes them particularly useful for the rapid generation of biomass (109). Another platform for growing conventional meat may be MuSC, which exhibit limited proliferative capacity. At the initial stages of cultivation, they can multiply intensively, but their potential is limited by cellular senescence, which is a limitation in the aspect of long-term cultivation. ESCs have unlimited proliferative capacity, although their maintenance in culture, especially in farm animals, requires specific culture conditions (89).

### 8.3 Differentiation potential

Another important criterion in the selection of appropriate cells that form the platform for culturing conventional meat is the ability to differentiate. ESC and iPSC are pluripotent, which means they have the highest differentiation potential, and they can give rise to various types of somatic cells, including muscle cells, fat cells, and supporting cells (110). Although their use is correlated with the necessity of monitoring differentiation pathways, the risk of undesired heterogeneity of the cell population, and the significant cost of growth factors and culture media. Furthermore, the use of stem cells may have some regulatory issues concerning the safety and genetic stability of these cells (111). MuSC, despite their limited pluripotency, have the ability to differentiate in a myogenic lineage, which ensures natural muscle regeneration mechanisms *in vivo*, and on the other hand, enables the production of highly functional muscle tissue. Despite several advantages, the use of MSC is fraught with several challenges. The main disadvantage is their limited proliferative capacity and, as their limited differentiation capacity (112). Accordingly, it is essential to develop strategies to maintain their “stemness” during expansion. MSCs are multipotent and differentiate into adipocytes and myocytes. Their main advantage is their significant proliferative capacity. In terms of cultured meat, MSCs are particularly valuable as a source of fat cells and a supporter of tissue development through the secretion of paracrine factors. Fibroblasts, although they have limited differentiation capacity, also play an important role in regulating the cellular microenvironment through ECM synthesis, which may improve the support function of newly formed muscle (113).

Collectively, the differentiation potential of these cells differs, which directly impacts their suitability for cultured meat production. Pluripotent cells possess the greatest cellular plasticity, while MuSC ensure the highest biocompatibility with native muscle tissue. However, the challenge remains to develop engineering strategies that enable precise, efficient, and reproducible control of this process on an industrial scale.

### 8.4 Scalability and design of bioprocesses

Scalability is a key criterion in conventional meat production, requiring simultaneous consideration of cell biological limitations and the engineering requirements of culture systems (114). One of the biggest challenges in the development of cultured meat is scaling up cell culture for industrial purposes. In large-volume bioreactors at the industrial scale, significant limitations arise related to oxygen transfer and nutrient diffusion, which can lead to heterogeneous conditions within the culture chamber affecting cell growth and differentiation (115). Furthermore, mixing, including the resulting stresses, can negatively affect adherent cells, reducing their viability. It is therefore crucial to design so-called stirred-tank or perfusion bioreactors. These can enable more efficient and effective production (115, 116).

Fibroblasts appear to have the highest compatibility with industrial conditions. In other words, they are characterized by high resistance to changes in pH, oxygen levels, and shear forces, as well as relatively low nutritional requirements. A distinguishing feature is the ability to produce ECM, which serves as a scaffold for future tissue and can positively influence the differentiation of other cells (117). Satellite cells are essential for the formation of native muscle tissue. It should also be noted that these cells are adhesive and, in suspension conditions they lose their proliferation capacity and enter a quiescent state. Consequently, microcarriers are used in bioreactors to create a growth surface while culturing them in volumetric systems. The microcarrier approach allows for increased cell density, although it requires additional technological applications, such as carrier aggregation and the need to remove or integrate them into the final product (118, 119). Pluripotent cells (ESCs/iPSCs) offer the greatest potential for scalability due to their unlimited proliferation capacity and adaptability to suspension culture. However, their use is hampered by high costs associated with the purchase of culture media, the need for precise control of differentiation, and regulatory complexity, which hinders their industrial implementation (120).

2D culture systems are mainly used in the research phase, and their scalability is limited by a low surface-to-volume ratio (117). The most promising approach is stirred-tank bioreactors, which enable control of process parameters and achieve high cell densities. Nevertheless, shear forces are a limitation, which can negatively impact sensitive cell populations (119). Furthermore, perfusion systems and solid beds have been developed, which allow for better control of culture conditions and higher cell densities, but are technologically more complex and more difficult to scale. Ongoing research pertaining to 3D systems based on scaffolds, which enable the replication of native tissue structure and improved organoleptic properties of the product, but their use is limited by high material costs and oxygen diffusion (121). When creating an effective bioprocess, many factors must be considered, including the appropriate cell type, its associated proliferative and differentiation properties, the selection of an appropriate culture medium, and control of physicochemical conditions. Automation and monitoring of the process are also crucial to ensure its repeatability and stability (114).

The scalability of conventional meat production is closely correlated with matching the appropriate biological properties of cells to the capabilities of bioreactor systems. Fibroblasts offer the greatest process simplicity, satellite cells ensure the highest biological quality of tissue, and pluripotent cells represent the most promising long-term solution.

### 8.5 Sensory significance and tissue composition

The organoleptic properties of meat, such as taste, aroma, juiciness, and texture, are the result of the complex interaction of many cell types and their metabolic products. Reproducing the representation of these cells in their appropriate proportions, spatial organization, and biological functions (122). MuSC are responsible for meat structure by differentiating into myocytes. Their degree of maturity and spatial arrangement directly influence the mechanical properties of the tissue, such as tenderness and elasticity. Adipocytes impart flavor and juiciness to farmed meat. Intramuscular fat (marbling) is correlated with the release of aromatic compounds during thermal processing. Furthermore, the lipid composition of adipocytes, dependent on factors such as the species and culture conditions, ultimately determines the flavor of the final product (123). Fibroblasts, produce collagen and other ECM components that influence meat structure. The amount and organization of collagen determine the tissue's hardness and structure, but excessive amounts can lead to increased stiffness. ESCs and iPSCs differentiate into all of the above-mentioned cell types, making them an adequate source for reproducing native muscle tissue. A significant limitation in the use of these cells is the difficulty in precisely controlling differentiation and maintaining stable and homogeneous cell populations. Inappropriate regulation of these processes can contribute to undesirable heterogeneity, which may negatively impact the organoleptic quality of the product (124).

Paracrine communication between cells, can influence their differentiation, metabolism, and spatial organization, ultimately impacting the final sensory properties of meat. As a result, the creation of these sensory characteristics requires the development of co-cultures and appropriate 3D systems, such as scaffolds, which allow for the reproduction of the native structure of muscle tissue through the appropriate arrangement of individual cell types. Another aspect still under investigation is the selection of appropriate media and the analysis of mechanical stimuli that can modulate the metabolic properties of cells and thus influence the taste and texture of meat (5). Thus, the organoleptic properties of conventional meat are the result of the interaction of muscle, fat, and support cells. Restoring native muscle tissue is one of the key challenges in the development of cultured meat technology and requires the integration of biological and engineering approaches.

### 8.6 Regulatory and ethical aspects

Regulatory and ethical aspects play a significant role in the development and commercialization of cultured meat. Apart from technological limitations, regulatory and food safety issues are also important. The production of cultivated meat requires quality control measures at every stage of production. Additionally, it is essential to monitor the genetic stability of cell lines and prevent any microbiological contamination of the production process (8, 125). Another significant limitation is the process of obtaining market authorization for cultivated meat from institutions such as the Food and Drug Administration (FDA) or the European Food Safety Authority (EFSA). These are regulatory bodies whose operations are quite complex and involve demonstrating the safety and stability of the production process. These challenges are a significant factor limiting the pace at which cultivated meat is brought to market. Furthermore, identifying potential risks arising from the novel nature of production processes and the sparse scientific data is important (126). Another challenge is the need to develop new adaptation methods and validate existing ones. The goal is to tailor these methods to different types of processes and the resulting materials. Furthermore, data collection and a better understanding of this modern technology could accelerate the evaluation process. Such risk assessment, along with access to reliable data, are key elements in the consumer market. These factors make any regulations time-consuming and also constitute a barrier that limits the pace of introducing cultured meat to the market (8).

The selection of the appropriate cell type has a direct impact not only on the efficiency of the process, but also on its social acceptability and the possibility of approval by regulatory bodies (110). Fibroblasts are among the most accepted cell types from a regulatory perspective. Furthermore, they are ubiquitous in animal organisms and their cultivation is straightforward. Their use falls within the framework of food safety assessment. MuSC are another acceptable cell type for conventional meat culture. They occur in native muscle tissue and are directly responsible for tissue regeneration *in vivo*. Interestingly, their use does not require genetic modification, which reduces regulatory implications and increases consumer acceptance (124). The use of ESCs and iPSCs is associated with much greater ethical controversies than previous cells, due to their origin, and the main challenges still include issues of genetic instability, uncontrolled differentiation, and potential tumorigenicity (127).

Another regulatory aspect is the degree of technological processing of the product. Platforms requiring the use of recombinant growth factors or genetic engineering are classified as more complex, which lengthens the approval process. In contrast, the use of primary cells involves minimal intervention and is perceived as more compliant with existing food regulations Ethical considerations also include the welfare of the animals from which these cells are obtained. Cultured meat is an alternative that reduces animal use (128).

Cells with a low degree of modification, such as fibroblasts and satellite cells, are the most beneficial. However, ESC and iPSC cells, despite their pluripotency, require further research and development of regulatory frameworks to be widely used in the production of cultured meat.

## 9 CRISPR-based cell engineering

CRISPR/Cas systems have emerged as versatile and highly efficient tools for targeted genome engineering, enabling precise manipulation of cellular phenotypes relevant to cultured meat production. The canonical CRISPR/Cas9 system employs a single-guide RNA (sgRNA) to direct the Cas9 to a complementary genomic locus adjacent to a protospacer adjacent motif (PAM), resulting in a double-strand break (DSB) (129). Subsequent repair is conducted though non-homologous end joining (NHEJ) typically introducing insertions or deletions, or homology-directed repair (HDR) enabling precise sequence inserting in the presence of a donor template (130). Alternative CRISPR systems, such as Cas12a, exhibit distinct PAM requirements and generate staggered DNA breaks. Cas12a system recognizes a T-rich PAM, while Cas9 system is dependent on G-containing sequences. Cas12a is often advantageous because of its effectiveness in AT rich genomes (131). Cas9 produces blunt-ended DSB, approximately 3 bp upstream of the PAM, whereas Cas12a generates staggered cuts with 5′ overhangs distal to the PAM, which can enhance directional DNA insertion efficiency in knock-in applications (132). Recently, base-editing technologies have been developed that couple Cas nucleases with deaminases, allowing single-nucleotide conversion without inducing DSBs. It enables efficient, targeted conversion of individual nucleotides within a limited editing window, making them well suited for rapid correction of point mutations. In contrast, prime-editing are more versatile, supporting all twelve possible base substitutions as well as small insertions and deletions through a “search-and-replace” mechanism (133). These advancements collectively provide a comprehensive molecular toolkit for engineering cell lines optimized for *in vitro* meat production.

One of the primary applications of CRISPR/Cas technology in cultured meat systems involves enhancing the proliferative capacity of muscle progenitor cells, including satellite cells and myoblasts. Sustained proliferation without loss of differentiation potential is critical for scalable biomass production. CRISPR-mediated disruption of senescence-associated pathways, including those involving CDKN1A (p21) and CDKN2A (p16), has been shown to alleviate cellular senescence and improve proliferative capacity in human muscle progenitor cells (134, 135). Similarly, CRISPR-mediated activation or repression of key signaling pathways, including PI3K/AKT/mTOR and Wnt/β-catenin, has the potential to promote proliferation while preserving myogenic potential, as these pathways are central regulators of cell growth and stem cell function (136–138). In this context, CRISPR platforms provide additional layers of transcriptional control, which may be advantageous for proliferative dynamics.

Precise regulation of myogenic and adipogenic differentiation is another critical determinant of cultured meat quality, as the balance between muscle fibers and intramuscular fat directly influences texture, flavor, and nutritional value. CRISPR/Cas9 was used by Higashioka et al. (139) to generate myogenin-mutated human iPS, describing importance of its cooperation with other myogenic factors from myogenic regulatory factors (MRF) gene family (139). CRISPR base-editing has found its application in medicine, where a mutation in a gene responsible for muscular dystrophy was edited. Localization and subsequent replacement of the defective nucleotide resulted in a repair of the cell phenotype, which was then transferred to an *in vivo* mouse model (140). Prime-editing technology has been applied in similar clinical evidence. Extensive nature of prime-editing technology has led to correcting reading frames of dystrophy related genes restoring dystrophin synthesis and dystrophin-β-dystroglycan linkages in muscle cell populations (141). Although these reports are not directly related to meat production, they demonstrate the scale of possibilities of using this technology in the food industry.

One of the most prominent targets is the myostatin gene (MSTN), a negative regulator of muscle growth. CRISPR/Cas-mediated disruption of MSTN removes intrinsic inhibitory constraints on myogenesis, resulting in enhanced proliferation and differentiation of muscle cells. Experimental approaches combining MSTN knockout with advanced tissue engineering strategies, such as 3D bioprinting, have demonstrated improved muscle fiber alignment and tissue maturation, yielding constructs that more closely resemble native muscle architecture (142, 143). CRISPR-mediated engineering also facilitates precise control over myogenic differentiation programs through manipulation of myogenic regulatory factors, including MYOD1 and MYOG. These transcription factors play central roles in muscle lineage specification and maturation, and their targeted modulation enables improved formation and alignment of myotubes in engineered tissues (142). Another important aspect of cultivated meat production is the adaptation of cells to the physicochemical conditions of large-scale bioreactors, particularly hypoxic environments associated with high-density cultures. Targeting hypoxia-inducible factors such as HIF1A via CRISPR-based approaches has been proposed to enhance cellular tolerance to low oxygen levels, thereby improving survival and metabolic efficiency under industrial cultivation conditions (144).

Despite these advances, several technical challenges remain in the application of CRISPR/Cas systems to cultured meat engineering. Off-target effects, resulting from imperfect sgRNA specificity, can lead to unintended genomic alterations that may compromise cell function or stability (145, 146). The use of high-fidelity Cas variants, improved sgRNA design algorithms, and orthogonal nucleases has partially mitigated these concerns, although comprehensive genome-wide validation remains necessary for industrial applications.

Collectively, CRISPR/Cas-based genome engineering provides a powerful and adaptable platform for optimizing cellular systems used in cultured meat production. Through precise modulation of proliferation, differentiation, metabolic pathways, and structural properties, these technologies enable the rational design of cell lines tailored for scalable and high-quality biomass generation.

## 10 Conclusion

Currently, the production of cultured meat is moving from proof-of-concept research to industrial implementation, where the choice of cell types plays a significant role. Fibroblasts offer scalability benefits however, they cannot replicate the native structure of muscle tissue. Satellite cells, especially in co-culture with adipogenic cells, represent a biologically significant approach and are increasingly preferred for the production of alternative meat. New methodologies, such as genetic engineering, immortalization, and transdifferentiation, have significant potential.

Future progress in this field will likely depend on hybrid systems integrating multiple cell types, as well as advances in bioprocessing, media optimization, and regulatory harmonization.

## References

[1] Alexandratos N., and Bruinsma, J. (2012). World agriculture towards 2030/2050: the 2012 revision. ESA Working paper No. 12-03. Rome: FAO. Available online at: https://www.fao.org/4/ap106e/ap106e.pdf (Accessed January 15, 2026).

[2] Wang L Li G Li X Zhang Y Liu G Xie M . Emerging materials in cultivated meat: engineering sustainable food solutions–a review. Adv Funct Mater. (2024) 35:2413316. doi: 10.1002/adfm.202413316

[3] Bryant C Barnett J. Consumer acceptance of cultured meat: an updated review (2018-2020). Applied Sciences (Switzerland). (2020) 10:5201. doi: 10.3390/app10155201

[4] Liu J Ellies-Oury MP Chriki S Isabelle L Grzegorz P Jerzy W . Contributions of tenderness, juiciness and flavor liking to overall liking of beef in Europe. Meat Sci. (2020) 168:108190. doi: 10.1016/j.meatsci.2020.108190 32450455

[5] Lee M Park S Choi B Choi W Lee H Lee JM . Cultured meat with enriched organoleptic properties by regulating cell differentiation. Nat Commun. (2024) 15:77. doi: 10.1038/s41467-023-44359-9 38167486 PMC10762223

[6] Folmer D Simmons C Zhang J Stice S Overbey K Hice S. Cell culture consultation (CCC) 000008, cultured Sus scrofa domesticus cell material. Food Drug Administr. (2022). Available online at: https://www.fda.gov/media/185744/download (Accessed December 18, 2025).

[7] Chriki S Ellies-Oury MP Hocquette JF. Is “cultured meat” a viable alternative to slaughtering animals and a good comprise between animal welfare and human expectations?. Anim Front. (2022) 12:35–42. Published 2022 Mar. 17. doi: 10.1093/af/vfac002 PMC8929989 35311183

[8] Khan I Sun J Liang W Li R Cheong KL Qiu Z . Innovations, challenges, and regulatory pathways in cultured meat for a sustainable future. Foods. (2025) 14:3183. doi: 10.3390/foods14183183 41008156 PMC12469421

[9] Monaco A. A perspective on the regulation of cultivated meat in the European Union. NPJ Science of Food. (2025) 9:21. doi: 10.1038/s41538-025-00384-0 39922830 PMC11807164

[10] Zhao W Li Z Ma S Chen W Wan Z Zhu L . Identification of pro-fibrotic cellular subpopulations in fascia of gluteal muscle contracture using single-cell RNA sequencing. J Transl Med. (2025) 23:192. doi: 10.1186/s12967-024-05889-y 39962491 PMC11834283

[11] Mendias CL. Fibroblasts take the centre stage in human skeletal muscle regeneration. J Physiol. (2017) 595:5005. doi: 10.1113/JP274403 28585689 PMC5538237

[12] Mackey AL Magnan M Chazaud B Kjaer M. Human skeletal muscle fibroblasts stimulate in vitro myogenesis and in vivo muscle regeneration. J Physiol. (2017) 595:5115–27. doi: 10.1113/JP273997 28369879 PMC5538230

[13] Tomaz da Silva M Joshi AS Kumar A. Fibroblast growth factor–inducible 14 regulates satellite cell self-renewal and expansion during skeletal muscle repair. JCI Insight. (2025). 10:e187825. doi: 10.1172/jci.insight.187825 PMC11949035 39874107

[14] Ben-Arye T Levenberg S. Tissue engineering for clean meat production. Front Sustainable Food Systems. (2019) 3: 2019.00046. doi: 10.3389/fsufs.2019.00046

[15] Hauser M Zirman A Rak R Nachman I. Challenges and opportunities in cell expansion for cultivated meat. Front Nutr. (2024) 11:1315555. doi: 10.3389/fnut.2024.1315555 38385010 PMC10879929

[16] Jackson M Krassowska A Gilbert N Chevassut T Forrester L Ansell J . Severe global DNA hypomethylation blocks differentiation and induces histone hyperacetylation in embryonic stem cells. Mol Cell Biol. (2004) 24:8862–71. doi: 10.1128/MCB.24.20.8862-8871.2004 15456861 PMC517875

[17] Romagnoli C Iantomasi T Brandi ML. Available in vitro models for human satellite cells from skeletal muscle. Int J Mol Sci. (2021) 22:13221. doi: 10.3390/ijms222413221 34948017 PMC8706222

[18] O'Neill EN Ansel JC Kwong GA Plastino ME Nelson J Baar K aet al. Spent media analysis suggests cultivated meat media will require species and cell type optimization. NPJ Sci Food. (2022) 6:46. doi: 10.1038/s41538-022-00157-z 36175443 PMC9523075

[19] Mafruchati M Othman NH. Fibroblast test cells of embryo of super java chicken as an indicator to test toxicity and malignancy. Heliyon. (2023) 9:e22349. doi: 10.1016/j.heliyon.2023.e22349 38125449 PMC10730434

[20] Pasitka L Cohen M Ehrlich A Gildor B Reuveni E Ayyash M . Spontaneous immortalization of chicken fibroblasts generates stable, high-yield cell lines for serum-free production of cultured meat. Nature Food. (2023) 4:35–50. doi: 10.1038/s43016-022-00658-w 37118574

[21] Pasitka L Wissotsky G Ayyash M Yarza N Rosoff G Kaminker R . Empirical economic analysis shows cost-effective continuous manufacturing of cultivated chicken using animal-free medium. Nature Food. (2024) 5:693–702. doi: 10.1038/s43016-024-01022-w 39179871

[22] Mullen N Park N Jones C Bowman T Bignone P Santo VE . In vitro avian food product (United States Patent No. US20200392461A1). (2020). Available online at: https://patents.google.com/patent/US20200392461A1/en (Accessed January 15, 2026).

[23] Foster D. N. Foster L. K. (1997). Immortalized cell lines for virus growth (United States Patent No. US5672485A). Available online at: https://patents.google.com/patent/US5672485A/en (Accessed November 22, 2025).

[24] Overbey K Folmer D Zhang J Kaneko K Stice S Fasano J . Cell culture consultation (CCC) 000001, cultured Gallus gallus cell material. Food Drug Administr. (2022). Available online at: https://agnetwest.com/wp-content/uploads/2023/06/GOOD-Meat-Cell-Culture-Consultation-000001-Submission-032120232_0.pdf (Accessed January 13, 2026).

[25] Watson E. GOOD Meat gets green light from FDA for cultivated meat, edges closer to commercialization in the US. AgFunderNews. (2023). Available online at: https://agfundernews.com/good-meat-gets-green-light-from-fda-for-cultivated-meat (Accessed December 18, 2025).

[26] Kim D-H Lee J Suh Y Cressman M Lee K. Research Note: adipogenic differentiation of embryonic fibroblasts of chicken, turkey, duck, and quail in vitro by medium containing chicken serum alone. Poult Sci. (2021) 100:101277. doi: 10.1016/j.psj.2021.101277 34198089 PMC8255238

[27] Kalgudde Gopal S. (2021). Adipocytes mobilize in response to injury without plasticity [Text.PhDThesis, Ludwig-Maximilians-Universität München]. Available online at: https://edoc.ub.uni-muenchen.de/28540/

[28] Sowa Y Kishida T Louis F Sawai S Seki M Numajiri T . Direct conversion of human fibroblasts into adipocytes using a novel small molecular compound: implications for regenerative therapy for adipose tissue defects. Cells. (2021) 10:605. doi: 10.3390/cells10030605 33803331 PMC8000077

[29] Kim S-H Kim C-J Lee E-Y Hwang Y-H Joo S-T. Chicken embryo fibroblast viability and trans-differentiation potential for cultured meat production across passages. Cells. (2024) 13:1734. doi: 10.3390/cells13201734 39451252 PMC11506350

[30] Huang H-K Hsueh K-K Liao Y-T Wu S-H Chou P-H Yeh S-H . Multilineage differentiation potential in the infant adipose- and umbilical cord-derived mesenchymal stem cells. Journal of the Chinese Medical Association. (2023) 86:1083–95. doi: 10.1097/JCMA.0000000000000990 37691559 PMC12718807

[31] Ma T Ren R Lv J Yang R Zheng X Hu Y . Transdifferentiation of fibroblasts into muscle cells to constitute cultured meat with tunable intramuscular fat deposition. Elife. (2024) 13:RP93220. doi: 10.7554/eLife.93220 38771186 PMC11108645

[32] Li Y Li C Zhou Q Liu X Qiao Y Xie T . Multiomics and cellular senescence profiling of aging human skeletal muscle uncovers maraviroc as a senotherapeutic approach for sarcopenia. Nat Commun. (2025) 16:6207. doi: 10.1038/s41467-025-61403-y 40617829 PMC12228793

[33] Pan Y Zhang Y Lu H Liu Z Ding S Li C . Molecular dynamics of immortalized chicken fibroblasts from adherent cultivation to carrier-free suspension culture. Food Bioscience. (2025) 73:107716. doi: 10.1016/j.fbio.2025.107716

[34] Contreras EJ Nagarajan A Bromberg BH Villegas MP Kaplan DL. Comparative transcriptomics of adherent and suspension chicken fibroblast cell lines for the optimization of cultivated meat processes. bioRxiv. (2025) p. 2025.09.02.673824. doi: 10.1101/2025.09.02.673824 41763754

[35] Tsuruwaka Y Shimada E. Reprocessing seafood waste: challenge to develop aquatic clean meat from fish cells. NPJ Sci Food. (2022) 6:7. doi: 10.1038/s41538-021-00121-3 35087061 PMC8795430

[36] Goswami M Ovissipour R Bomkamp C Nitin N Lakra W Post M . Cell-cultivated aquatic food products: emerging production systems for seafood. J Biol Eng. (2024) 18:43. doi: 10.1186/s13036-024-00436-1 39113103 PMC11304657

[37] Ikeda D Otsuka Y Kan-no N. Development of a novel Japanese eel myoblast cell line for application in cultured meat production. Biochem Biophy Res Com. (2024). 734:150784. doi: 10.1016/j.bbrc.2024.150784 39366176

[38] Yashwanth BS Pinto N Sathiyanarayanan A Rasal KD Sanjeeva S Saad M . In vitro protein expression profile of cultivated muscle cells from *Labeo rohita*. Sci Rep. (2025) 15:5859. doi: 10.1038/s41598-025-88900-w 39966484 PMC11836462

[39] Benjaminson MA Gilchriest JA Lorenz M. In vitro edible muscle protein production system (mpps): Stage 1, fish. Acta Astronaut. (2002) 51:879–89. doi: 10.1016/S0094-5765(02)00033-4 12416526

[40] Johnston EF Gillis TE. Transforming growth factor beta-1 (TGF-β1) stimulates collagen synthesis in cultured rainbow trout cardiac fibroblasts. J Exp Biol. (2017) 220:2645–53. doi: 10.1242/jeb.160093 28495868

[41] Rescan P-Y. Development of myofibres and associated connective tissues in fish axial muscle: Recent insights and future perspectives. Differentiation. (2019) 106:35–41. doi: 10.1016/j.diff.2019.02.007 30852471

[42] Chailom P Pattarakankul T Palaga T Hoven VP. Fish gelatin-hyaluronic acid scaffold for construction of an artificial three-dimensional skin model. ACS Omega. (2025) 10:8172–81. doi: 10.1021/acsomega.4c09708 40060871 PMC11886714

[43] Zhou H Loo LSW Ong FYT Lou X Wang J Myint MK . Cost-effective production of meaty aroma from porcine cells for hybrid cultivated meat. Food Chem. (2025) 473:142946. doi: 10.1016/j.foodchem.2025.142946 39864181

[44] Liu Q Xie L Chen W. Recombinant porcine FGF1 promotes muscle stem cell proliferation and mitochondrial function for cultured meat production. J Agric Food Chem. (2025) 73:2008–18. doi: 10.1021/acs.jafc.4c09215 39772551

[45] Su X Cui K Du S Li H Lu F Shi D . Efficient genome editing in cultured cells and embryos of Debao pig and swamp buffalo using the CRISPR/Cas9 system. In: Vitro Cellular and Developmental Biology-Animal. (2018) 54:375–83. doi: 10.1007/s11626-018-0236-8 29556895

[46] Xiang J Wang H Zhang Y Wang J Liu F Han X . LCDM medium supports the derivation of bovine extended pluripotent stem cells with embryonic and extraembryonic potency in bovine–mouse chimeras from iPSCs and bovine fetal fibroblasts. FEBS J. (2021) 288:4394–411. doi: 10.1111/febs.15744 33524211

[47] Recchia K Wathikthinnakon M Bressan FF Freude K. Generation of bovine iPSCs from fetal fibroblasts for in vitro myogenesis and cultured meat. Front Nutr. (2025) 12:1562981. doi: 10.3389/fnut.2025.1562981 40453727 PMC12124125

[48] Jeong D Seo JW Lee H Jung WK Park YH Bae H. Efficient myogenic/adipogenic transdifferentiation of bovine fibroblasts in a 3d bioprinting system for steak-type cultured meat production. Adv Sci. (2022) 9:e2202877. doi: 10.1002/advs.202202877 PMC9631076 36192168

[49] Ben-Arye T Shandalov Y Ben-Shaul S Landau S Zagury Y Ianovici I . Textured soy protein scaffolds enable the generation of three-dimensional bovine skeletal muscle tissue for cell-based meat. Nature Food. (2020) 1:210–20. doi: 10.1038/s43016-020-0046-5

[50] Defendi-Cho G Gould TM. In vitro culture of bovine fibroblasts using select serum-free media supplemented with *Chlorella vulgaris* extract. BMC Biotechnol. (2023) 23:4. doi: 10.1186/s12896-023-00774-w 36755248 PMC9909908

[51] Weber T Malakpour-Permlid A Chary A D'Alessandro V Haut L Seufert S . Fetal bovine serum: How to leave it behind in the pursuit of more reliable science. Front Toxicol. (2025) 7:1612903. doi: 10.3389/ftox.2025.1612903 40861932 PMC12371577

[52] Hillsley A Santos JE Rosales AM. A deep learning approach to identify and segment alpha-smooth muscle actin stress fiber positive cells. Scientific Rep. (2021) 11:21855. doi: 10.1038/s41598-021-01304-4 PMC8575943 34750438

[53] Sousa AM Liu T Guevara O Stevens JA Fanburg BL Gaestel M . Smooth muscle α-actin expression and myofibroblast differentiation by TGFβ are dependent upon MK2. J Cell Biochem. (2007) 100:1581. doi: 10.1002/jcb.21154 17163490 PMC2586991

[54] Morgan JE Partridge TA. Muscle satellite cells. Int J Biochem Cell Biol. (2003) 35:1151–6. doi: 10.1016/S1357-2725(03)00042-6 12757751

[55] Mauro A. Satellite cell of skeletal muscle fibers. J Cell Biol. (1961) 9:493–5. doi: 10.1083/jcb.9.2.493 PMC2225012 13768451

[56] Schultz E. Satellite cell proliferative compartments in growing skeletal muscles. Dev Biol. (1996) 175:84–94. doi: 10.1006/dbio.1996.0097 8608871

[57] Gonzalez ML Busse NI Waits CM Johnson SE. Satellite cells and their regulation in livestock. J Animal Sci. (2020) 98:skaa081. doi: 10.1093/jas/skaa081 PMC7193651 32175577

[58] Baraibar MA Hyzewicz J Rogowska-Wrzesinska A Bulteau A-L Prip-Buus C Butler-Browne G . Impaired energy metabolism of senescent muscle satellite cells is associated with oxidative modifications of glycolytic enzymes. Aging. (2016) 8:3375–89. doi: 10.18632/aging.101126 27922824 PMC5270674

[59] Renault V Thornell L-E Eriksson P-O Butler-Browne G Mouly V. Regenerative potential of human skeletal muscle during aging. Aging Cell. (2002) 1:132–9. doi: 10.1046/j.1474-9728.2002.00017.x 12882343

[60] Dumont N Bentzinger C Sincennes M-C Rudnicki M. Satellite cells and skeletal muscle regeneration. Compr Physiol. (2015) 5:1027–59. doi: 10.1002/j.2040-4603.2015.tb00646.x 26140708

[61] He Y Xie W Li H Jin H Zhang Y Li Y. Cellular senescence in sarcopenia: possible mechanisms and therapeutic potential. Front Cell Dev Biol. (2022) 9:793088. doi: 10.3389/fcell.2021.793088 35083219 PMC8784872

[62] Riquelme-Guzmán C Stout AJ Kaplan DL Flack JE. Unlocking the potential of cultivated meat through cell line engineering. iScience. (2024) 110877. doi: 10.1016/j.isci.2024.110877 39351194 PMC11440241

[63] Stout AJ Arnett MJ Chai K Guo T Liao L Mirliani AB . Immortalized bovine satellite cells for cultured meat applications. ACS Synth Biol. (2023) 12:1567–73. doi: 10.1021/acssynbio.3c00216 37146268

[64] Stout AJ Zhang X Letcher SM Rittenberg ML Shub M Chai KM . Engineered autocrine signaling eliminates muscle cell FGF2 requirements for cultured meat production. Cell Rep Sustain. (2024) 1:100009. doi: 10.1016/j.crsus.2023.100009

[65] Lepper C Conway SJ Fan C-M. Adult satellite cells and embryonic muscle progenitors have distinct genetic requirements. Nature. (2009) 460:627–31. doi: 10.1038/nature08209 19554048 PMC2767162

[66] Nunes OB da Buranello TW Farias FA Rosero J Recchia K . Can cell-cultured meat from stem cells pave the way for sustainable alternative protein? Cultured meat from iPSCs. Current Res Food Sci. (2025) 10:100979. doi: 10.1016/j.crfs.2025.100979 PMC11878651 40040753

[67] Skrivergaard S Rasmussen MK Therkildsen M Young JF. Bovine satellite cells isolated after 2 and 5 days of tissue storage maintain the proliferative and myogenic capacity needed for cultured meat production. Int J Mol Sci. (2021) 22:8376. doi: 10.3390/ijms22168376 34445082 PMC8395070

[68] Hathaway MR Hembree JR Pampusch MS Dayton WR. Effect of transforming growth factor beta-1 on ovine satellite cell proliferation and fusion. J Cell Physiol. (1991) 146:435–41. doi: 10.1002/jcp.1041460314 2022697

[69] Matschak TW Stickland NC. The growth of Atlantic salmon (*Salmo salar* L.) myosatellite cells in culture at two different temperatures. Experientia. (1995) 51:260–6. doi: 10.1007/BF01931109 7698290

[70] Seiliez I Gabillard J-C Skiba-Cassy S Garcia-Serrana D Gutiérrez J Kaushik S . An in vivo and in vitro assessment of TOR signaling cascade in rainbow trout (*Oncorhynchus mykiss*). Am J Physiol Regul, Integr Comp Physiol. (2008) 295:R329–335. doi: 10.1152/ajpregu.00146.2008 18434442

[71] Vélez EJ Lutfi E Jiménez-Amilburu V Riera-Codina M Capilla E Navarro I . IGF-I and amino acids effects through TOR signaling on proliferation and differentiation of gilthead sea bream cultured myocytes. General Comp Endocrinol. (2014) 205:296–304. doi: 10.1016/j.ygcen.2014.05.024 24882593

[72] Goswami M Pinto N Yashwanth BS Sathiyanarayanan A Ovissipour R. Development of a cell line from skeletal trunk muscle of the fish *Labeo rohita*. Cytotechnol. (2023) 75:349–61. doi: 10.1007/s10616-023-00581-3 PMC10299978 37389130

[73] Saad MK Yuen JSK Joyce CM Li X Lim T Wolfson TL . Continuous fish muscle cell line with capacity for myogenic and adipogenic-like phenotypes. Sci Rep. (2023) 13:5098. doi: 10.1038/s41598-023-31822-2 36991012 PMC10060565

[74] Rubio N Datar I Stachura D Kaplan D Krueger K. Cell-based fish: a novel approach to seafood production and an opportunity for cellular agriculture. Front Sustain Food Sys. (2019) 3. doi: 10.3389/fsufs.2019.00043

[75] Solhaug A Dowd GC Dayeh VR Sindre H Lee LEJ Bols NC. Improve your success with fish cell lines—small things that matter. In Vitro Cell Develop Biol-Animal. (2025). doi: 10.1007/s11626-025-01042-1 PMC13053418 40205252

[76] Montarras D Morgan J Collins C Relaix F Zaffran S Cumano A . Direct isolation of satellite cells for skeletal muscle regeneration. Science. (2005) 309:2064–7. doi: 10.1126/science.1114758 16141372

[77] Danoviz ME Yablonka-Reuveni Z. Skeletal muscle satellite cells: background and methods for isolation and analysis in a primary culture system. In:J. DiMario (, Ed.), *Methods in Molecular Biology*. Totowa, NJ: Humana Press. 798:21–52. doi: 10.1007/978-1-61779-343-1_2 PMC3325159 22130829

[78] Messmer T Klevernic I Furquim C Ovchinnikova E Dogan A Cruz H . A serum-free media formulation for cultured meat production supports bovine satellite cell differentiation in the absence of serum starvation. Nature Food. (2022) 3:74–85. doi: 10.1038/s43016-021-00419-1 37118488

[79] Hanga MP Ali J Moutsatsou P de la Raga FA Hewitt CJ Nienow A Wall I. Bioprocess development for scalable production of cultivated meat. Biotechnol Bioeng. (2020) 117:3029–39. doi: 10.1002/bit.27469 32568406

[80] Aguanno S Petrelli C Di Siena S De Angelis L Pellegrini M Naro F. A Three-dimensional culture model of reversibly quiescent myogenic cells. Stem Cells Int. (2019) 2019:7548160. doi: 10.1155/2019/7548160 31827532 PMC6885280

[81] Bodiou V Cristini N De Cristofaro L Pareek T Rajagopal V Verrougstraete L . Process intensification of cultivated meat production through microcarrier addition strategy optimisation. Sci Rep. (2025) 15:14080. doi: 10.1038/s41598-025-97813-7 40269015 PMC12019398

[82] Kadi F Ponsot E. The biology of satellite cells and telomeres in human skeletal muscle: Effects of aging and physical activity. Scand J Med Sci Sports. (2010) 20:39–48. doi: 10.1111/j.1600-0838.2009.00966.x 19765243

[83] Piedrahita JA Anderson GB BonDurant RH. On the isolation of embryonic stem cells: Comparative behavior of murine, porcine and ovine embryos. Theriogenology. (1990) 34:879–901. doi: 10.1016/0093-691X(90)90559-C 16726890

[84] Choi K-H Lee D-K Kim SW Woo S-H Kim D-Y Lee C-K. Chemically defined media can maintain pig pluripotency network in Vitro. Stem Cell Reports. (2019) 13:221–34. doi: 10.1016/j.stemcr.2019.05.028 31257130 PMC6626979

[85] Kinoshita M Kobayashi T Planells B Klisch D Spindlow D Masaki H . Pluripotent stem cells related to embryonic disc exhibit common self-renewal requirements in diverse livestock species. Development. (2021) 148:dev199901. doi: 10.1242/dev.199901 34874452 PMC8714072

[86] Zhu H Wu Z Ding X Post MJ Guo R Wang J . Production of cultured meat from pig muscle stem cells. Biomaterials. (2022) 287:121650. doi: 10.1016/j.biomaterials.2022.121650 35872554

[87] Sukparangsi W Thongphakdee A Intarapat S. Avian embryonic culture: a perspective of in ovo to ex ovo and in vitro studies. Front Physiol. (2022) 13:903491. doi: 10.3389/fphys.2022.903491 35651873 PMC9150135

[88] Costa ML Jurberg AD Mermelstein C. The role of embryonic chick muscle cell culture in the study of skeletal myogenesis. Front Physiol. (2021) 12:668600. doi: 10.3389/fphys.2021.668600 34093232 PMC8173222

[89] Reiss J Robertson S Suzuki M. Cell Sources for Cultivated Meat: applications and considerations throughout the production workflow. Int J Mol Sci. (2021) 22:7513. doi: 10.3390/ijms22147513 34299132 PMC8307620

[90] Bellani CF Ajeian J Duffy L Miotto M Groenewegen L Connon CJ. Scale-up technologies for the manufacture of adherent cells. Front Nutr. (2020) 7:575146. doi: 10.3389/fnut.2020.575146 33251241 PMC7672005

[91] Tsai AC Pacak CA. Bioprocessing of human mesenchymal stem cells: from planar culture to microcarrier-based bioreactors. Bioengineering. (2021) 8:96. doi: 10.3390/bioengineering8070096 34356203 PMC8301102

[92] Altmann B Grün C Nies C Gottwald E. Advanced 3D cell culture techniques in micro-bioreactors, part II: systems and applications. Processes. (2021) 9:21. doi: 10.3390/pr9010021

[93] Shaikh S Lee EJ Ahmad K Ahmad SS Chun HJ Lim JH . Cell types used for cultured meat production and the importance of myokines. Foods. (2021) 10:2318. doi: 10.3390/foods10102318 34681367 PMC8534705

[94] Samandari M Saeedinejad F Quint J Chuah SXY Farzad R Tamayol A. Repurposing biomedical muscle tissue engineering for cellular agriculture: challenges and opportunities. Trends Biotechnol. (2023) 41:887–906. doi: 10.1016/j.tibtech.2023.02.002 36914431 PMC11412388

[95] Fonseca LN Bolívar-Moná S Agudelo T Beltrán LD Camargo D Correa N . Cell surface markers for mesenchymal stem cells related to the skeletal system: a scoping review. Heliyon. (2023) 9:e13464. doi: 10.1016/j.heliyon.2023.e13464 36865479 PMC9970931

[96] Heyman E Meeremans M Van Poucke M Peelman L Devriendt B De Schauwer C. Validation of multiparametric panels for bovine mesenchymal stromal cell phenotyping. Cytometry Part A. (2023) 103:744–55. doi: 10.1002/cyto.a.24737 37173856

[97] Thrower T Riley SE Lee S Esteves CL Donadeu FX. A unique spontaneously immortalised cell line from pig with enhanced adipogenic capacity. (2022). NPJ Sci Food. 9:52. doi: 10.1038/s41538-025-00413-y PMC12010005 40254637

[98] Haach V Silveira KRD Peixoto M de Sá APP Gressler V Feddern V . Establishment of chicken muscle and adipogenic cell cultures for cultivated meat production. Front Nutr. (2025) 12:1648935. doi: 10.3389/fnut.2025.1648935 41158655 PMC12554555

[99] Xue T Zheng H Zhao Z Wang J Li Y Wang S . Establishment and characterization of a continuous goldfish muscle stem cell line for cell-cultured fish meat production. Aquaculture. (2025) 606:742599. doi: 10.1016/j.aquaculture.2025.742599

[100] Yuen JSK Stout AJ Kawecki NS Letcher SM Theodossiou SK Cohen JM . Perspectives on scaling production of adipose tissue for food applications. Biomaterials. (2021) 280:121273. doi: 10.1016/j.biomaterials.2021.121273 34933254 PMC8725203

[101] Ying Ping L. Biological properties of adipose-derived stem cells (ADSCs) and bone marrow stem cells (BMSCs). Ping LY. Biological properties of adipose-derived stem cells (ADSCs) and bone marrow stem cells (BMSCs). J Clin Med Img. (2024) 7:1–7.

[102] Xuan Z Peng Q Borsuk M Prasad R Zachar V Das SK . Development of a sustainable strategy for cultured fat production based on serum-free 3D culture of bovine adipose stem cells. Sci Rep. (2025) 15:44793. doi: 10.1038/s41598-025-28441-4 41461685 PMC12749105

[103] Klatt A Wollschlaeger JO Albrecht FB Rühle S Holzwarth LB Hrenn H . Dynamically cultured, differentiated bovine adipose-derived stem cell spheroids as building blocks for biofabricating cultured fat. Nature Com. (2024) 15:9107. doi: 10.1038/s41467-024-53486-w PMC11496621 39438462

[104] Han JH Jang SW Kim YR Jang H Shim KS Choi HW. The fibronectin concentration that optimally maintains porcine satellite cells. Animal Biosci. (2023) 36:1889–97. doi: 10.5713/ab.23.0108 PMC10623030 37592381

[105] Sugii S Wong CYQ Lwin AKO Chew LJM. Alternative fat: redefining adipocytes for biomanufacturing cultivated meat. Trends Biotechnol. (2023) 41:686–700. doi: 10.1016/j.tibtech.2022.08.005 36117023

[106] Oki Y Hagiwara R Matsumaru T Kano K. Effect of volatile fatty acids on adipocyte differentiation in bovine dedifferentiated fat (DFAT) cells *in vitro*. Genes Cells : Devote Molecul Cell Mec. (2022) 27:5–13. doi: 10.1111/gtc.12903 34695306

[107] Peng X Song T Hu X Zhou Y Wei H Peng J . (2015). Phenotypic and functional properties of porcine dedifferentiated fat cells during the long-term culture. In Vitro BioMed Res Int. (2015). doi: 10.1155/2015/673651 PMC4450286 26090433

[108] Song J Lv Z Guo Y. Research advances in intramuscular fat deposition and chicken meat quality: genetics and nutrition. J Anim Sci Biotechnol. (2025) 16:100. doi: 10.1186/s40104-025-01234-5 40665461 PMC12265352

[109] Benny A Pandi K Upadhyay R. Techniques, challenges and future prospects for cell-based meat. Food Sci Biotechnol. (2022) 31:1225. doi: 10.1007/s10068-022-01136-6 35992324 PMC9385919

[110] Chehelgerdi M Behdarvand Dehkordi F Chehelgerdi M Kabiri H Salehian-Dehkordi H Abdolvand M . Exploring the promising potential of induced pluripotent stem cells in cancer research and therapy. Mol Cancer. (2023) 22:189. doi: 10.1186/s12943-023-01873-0 38017433 PMC10683363

[111] Yin H Price F Rudnicki MA. Satellite cells and the muscle stem cell niche. Physiol Rev. (2013) 93:23–67. doi: 10.1152/physrev.00043.2011 23303905 PMC4073943

[112] Ding S Swennen GNM Messmer T Gagliardi M Molin DGM Li C . Maintaining bovine satellite cells stemness through p38 pathway. Sci Rep. (2018) 8:10808. doi: 10.1038/s41598-018-28746-7 30018348 PMC6050236

[113] Jaime-Rodríguez M Cadena-Hernández AL Rosales-Valencia LD Padilla-Sánchez JM Chavez-Santoscoy RA. Are genetic drift and stem cell adherence in laboratory culture issues for cultivated meat production? Front Nutr. (2023) 10:1189664. doi: 10.3389/fnut.2023.1189664 37701376 PMC10493286

[114] Gu H Kong Y Huang D Wang Y Raghavan V Wang J. Scaling cultured meat: challenges and solutions for affordable mass production. Comprehensive Rev Food Sci Food Safety. (2025) 24:e70221. doi: 10.1111/1541-4337.70221 PMC12241508 40635127

[115] Ngwa CJ Chiu KH Brenner KJ Rodrigues C Peršin Z Vajda J . Bioreactor parameters and systems for cultured meat production. Future Foods. (2025) 12:100796. doi: 10.1016/j.fufo.2025.100796

[116] Fang Z Lyu J Li J Li C Zhang Y Guo Y . Application of bioreactor technology for cell culture-based viral vaccine production: present status and future prospects. Front Bioeng Biotechnol. (2022) 10:921755. doi: 10.3389/fbioe.2022.921755 36017347 PMC9395942

[117] Bhat ZF Kumar S Fayaz H. In vitro meat production: Challenges and benefits over conventional meat production. J Integr Agric. (2015) 14:241–8. doi: 10.1016/S2095-3119(14)60887-X

[118] Bodiou V Moutsatsou P Post MJ. Microcarriers for upscaling cultured meat production. Front Nutr. (2020) 7:10. doi: 10.3389/fnut.2020.00010 32154261 PMC7045063

[119] Kulus M Jankowski M Kranc W Golkar Narenji A Farzaneh M Dziegiel P . Bioreactors, scaffolds and microcarriers and in vitro meat production-current obstacles and potential solutions. Front Nutr. (2023) 10. doi: 10.3389/fnut.2023.1225233 PMC10513094 37743926

[120] Post MJ Levenberg S Kaplan DL Genovese N Fu J Bryant CJ . Scientific, sustainability and regulatory challenges of cultured meat. Nature Food.. (2020) 403–15. doi: 10.1038/s43016-020-0112-z

[121] Mirshafiei M Rashedi H Yazdian F Rahdar A Baino F. Advancements in tissue and organ 3D bioprinting: Current techniques, applications, and future perspectives. Materials and Design. (2024) 240:112853. doi: 10.1016/j.matdes.2024.112853

[122] Hwang YH Lee EY Lim HT Joo ST. Multi-omics approaches to improve meat quality and taste characteristics. Food Sci Animal Res. (2023) 43:1067. doi: 10.5851/kosfa.2023.e63 PMC10636221 37969318

[123] Moreira A Müller M Costa PF Kohl Y. Advanced In vitro lung models for drug and toxicity screening: the promising role of induced pluripotent stem cells. Advanced Biol. (2022) 6:2101139. doi: 10.1002/adbi.202101139 34962104

[124] Plikus MV Wang X Sinha S Forte E Thompson SM Herzog EL . Fibroblasts: origins, definitions, and functions in health and disease. Cell. (2021) 184:3852. doi: 10.1016/j.cell.2021.06.024 34297930 PMC8566693

[125] Zandonadi RP Ramos MC Elias FTS Guimarães NS. Global insights into cultured meat: Uncovering production processes, potential hazards, regulatory frameworks, and key challenges—A scoping review. Foods. (2025) 14:129. doi: 10.3390/foods14010129 39796419 PMC11720233

[126] Kirsch M Morales-Dalmau J Lavrentieva A. Cultivated meat manufacturing: technology, trends, and challenges. Eng Life Sci. (2023) 23:e2300227. doi: 10.1002/elsc.202300227 38089567 PMC10711323

[127] Moy AB Kamath A Ternes S Kamath J. The challenges to advancing induced pluripotent stem cell-dependent cell replacement therapy. Medical Research Archives. (2023) 11:4784. doi: 10.18103/mra.v11i11.4784 38188933 PMC10768945

[128] Sprink T Eriksson D Schiemann J Hartung F. Regulatory hurdles for genome editing: process- vs. product-based approaches in different regulatory contexts. Plant Cell Rep. (2016) 35:1493. doi: 10.1007/s00299-016-1990-2 27142995 PMC4903111

[129] Mengstie MA Wondimu BZ. Mechanism and applications of CRISPR/Cas-9-mediated genome editing. Biologics : Targets and Therapy. (2021) 15:353–353. doi: 10.2147/BTT.S326422 34456559 PMC8388126

[130] Fritsche S Reinfurt A Fronek F Steiger MG. NHEJ and HDR can occur simultaneously during gene integration into the genome of *Aspergillus niger*. Fungal Biology and Biotechnology 2024 11:1 11. (2024) 10. doi: 10.1186/s40694-024-00180-7 PMC11301975 39103967

[131] Schubert MS Thommandru B Woodley J Turk R Yan S Kurgan G . Optimized design parameters for CRISPR Cas9 and Cas12a homology-directed repair. Scientific Reports 2021 11:1 11. (2021) 19482. doi: 10.1038/s41598-021-98965-y PMC8484621 34593942

[132] Chew YP Ferenczi A Dannay M Ponce-Lilly C Kovac A Tóth D . Enhancing CRISPR/Cas-mediated gene knockout with short non-homologous oligonucleotides. Plant Biotechnol J. (2026). 10.1111/pbi.70548 PMC13205664 41725298

[133] Gupta PK Kumar S. Third-generation novel technologies for gene editing. Trends Biotechnol. (2025) 44:633–47. doi: 10.1016/j.tibtech.2025.07.012 40738762

[134] Li HY Wang M Jiang X Jing Y Wu Z He Y . CRISPR screening uncovers nucleolar RPL22 as a heterochromatin destabilizer and senescence driver. Nucleic Acids Res. (2024) 52:11481–99. doi: 10.1093/nar/gkae740 39258545 PMC11514463

[135] Moustogiannis A Philippou A Taso O Zevolis E Pappa M Chatzigeorgiou A . The effects of muscle cell aging on myogenesis. Int J Mol Sci. (2021) 22:3721–3721. doi: 10.3390/ijms22073721 33918414 PMC8038215

[136] Cui S Li L Yu RT Downes M Evans RM Hulin JA . β-Catenin is essential for differentiation of primary myoblasts via cooperation with MyoD and α-catenin. Development (Cambridge, England) (2019) 146:dev167080–dev167080. doi: 10.1242/dev.167080 30683662 PMC6451316

[137] Liu J Xiao Q Xiao J Niu C Li Y Zhang X . Wnt/β-catenin signalling: Function, biological mechanisms, and therapeutic opportunities. Signal Transduction and Targeted Therapy. (2022) 7:3. doi: 10.1038/s41392-021-00762-6 34980884 PMC8724284

[138] Madsen RR. PI3K in stemness regulation: from development to cancer. Biochem Soc Trans. (2020) 48:301–15. doi: 10.1042/BST20190778 32010943 PMC7054754

[139] Higashioka K Koizumi N Sakurai H Sotozono C Sato T. Myogenic differentiation from MYOGENIN-Mutated Human iPS Cells by CRISPR/Cas9. Stem Cells Int. (2017) 2017:9210494–9210494. doi: 10.1155/2017/9210494 28473859 PMC5394914

[140] Escobar H Krause A Keiper S Kieshauer J Müthel S de Paredes MG . Base editing repairs an SGCA mutation in human primary muscle stem cells. JCI Insight. (2021) 6:e145994. doi: 10.1172/jci.insight.145994 33848270 PMC8262330

[141] Wang Q Capelletti S Liu J Janssen JM Gonçalves MAFV. Selection-free precise gene repair using high-capacity adenovector delivery of advanced prime editing systems rescues dystrophin synthesis in DMD muscle cells. Nucleic Acids Res. (2024) 52:2740–2740. doi: 10.1093/nar/gkae057 38321963 PMC11648982

[142] Eom K-H Jeong D Choi J-Y Gim G-M Yum S-Y Jin S . MSTN knockout enhances the production of MYOD1-mediated steak-type cultivated meat. J Anim Sci Biotechnol. (2025) 16:41. doi: 10.1186/s40104-025-01173-1 40065420 PMC11895244

[143] Zhou S Kalds P Luo Q Sun K Zhao X Gao Y . Optimized Cas9:sgRNA delivery efficiently generates biallelic MSTN knockout sheep without affecting meat quality. BMC Genomics. (2022) 23:348. doi: 10.1186/s12864-022-08594-6 35524183 PMC9078021

[144] Jain IH Calvo SE Markhard AL Skinner OS To T-L Ast T Mootha VK. Genetic screen for cell fitness in high or low oxygen highlights mitochondrial and lipid metabolism. Cell. (2020) 181:716–27. doi: 10.1016/j.cell.2020.03.029 32259488 PMC7293541

[145] Guo C Ma X Gao F Guo Y. Off-target effects in CRISPR/Cas9 gene editing. Front Bioeng Biotechnol. (2023) 11:1143157. doi: 10.3389/fbioe.2023.1143157 36970624 PMC10034092

[146] Karimi MA Paryan M Behrouzian Fard G Sadeghian H Zarrinfar H Hosseini Bafghi M. Challenges and opportunities in the application of CRISPR-Cas9: a review on genomic editing and therapeutic potentials. Med Prin Prac (2025) 35:1–17. doi: 10.1159/000547334 PMC12503735 40675140

[147] Fan Z Liu L Huang X Zhao Y Zhou L Wang D Wei J. Establishment and growth responses of Nile tilapia embryonic stem-like cell lines under feeder-free condition. Dev Growth Differ. (2017) 59:83–93. doi: 10.1111/dgd.12341 28230233

[148] Keefer CL Pant D Blomberg L Talbot NC. Challenges and prospects for the establishment of embryonic stem cell lines of domesticated ungulates. Anim Reprod Sci. (2007) 98:147–68. doi: 10.1016/j.anireprosci.2006.10.009 17097839

[149] Post MJ. Cultured meat from stem cells: challenges and prospects. Meat Sci. (2012) 92:297–301. doi: 10.1016/j.meatsci.2012.04.008 22543115

[150] Vilarino M Alba Soto D Soledad Bogliotti Y Yu L Zhang Y Wang C . Derivation of sheep embryonic stem cells under optimized conditions. Reproduction (2020) 160:761–72. doi: 10.1530/REP-19-0606 33065542

[151] Sadiq IZ Abubakar FS Katsayal BS Ibrahim B Adamu A Usman MA . Stem cells in regenerative medicine: unlocking therapeutic potential through stem cell therapy, 3D bioprinting, gene editing, and drug discovery. Biome Eng Adv. (2025) 9:100172. doi: 10.1016/j.bea.2025.100172

[152] Kim D Roh S. Strategy to establish embryo-derived pluripotent stem cells in cattle. Int J Mol Sci. (2021) 22:5011. doi: 10.3390/ijms22095011 34065074 PMC8125899

[153] Ciarlo CA Zon LI. Embryonic cell culture in zebrafish. Methods Cell Biol. (2016) 133:1–10. doi: 10.1016/bs.mcb.2016.02.010 27263406 PMC6020034

[154] Chen Z Yu X Ke M Li H Jiang Y Zhang P . Human embryonic stem cell-derived cardiovascular progenitor cells stimulate cardiomyocyte cell cycle activity via activating the PI3K/Akt pathway. J Mol Cell Cardiol. (2024) 197:5-10. doi: 10.1016/j.yjmcc.2024.10.002 39393445

[155] Yang R Fei Z Wang L Tang H Sun W Li M . Highly efficient isolation and 3D printing of fibroblasts for cultured meat production. Front Sustain Food Syst. (2024) 8:1358862. doi: 10.3389/fsufs.2024.1358862

[156] Bogliotti YS Wu J Vilarino M Okamura D Soto DA Zhong C . Efficient derivation of stable primed pluripotent embryonic stem cells from bovine blastocysts. Proc Natl Acad Sci U S A. (2018) 115:2090–5. doi: 10.1073/pnas.1716161115 29440377 PMC5834688

[157] Dorota A Maryniak N Mariankowska A Milczarek C Dorota M Zywiec W . Induced Pluripotent Stem Cells (iPSC) and their use in disease modeling. Cureus. (2025) 17:e93999. doi: 10.7759/cureus.93999 41210051 PMC12590075

[158] Navarro M Laiz-Quiroga L Blüguermann C Mutto A. Livestock embryonic stem cells for reproductive biotechniques and genetic improvement. Anim Reprod. (2024) 21:e20240029. doi: 10.1590/1984-3143-AR2024-0029 39175999 PMC11340801

[159] Hwang WY Fu Y Reyon D Maeder ML Tsai SQ Sander JD . Efficient genome editing in zebrafish using a CRISPR-Cas system. Nat Biotechnol. (2013) 31:227–9. doi: 10.1038/nbt.2501 23360964 PMC3686313

[160] Auer TO Duroure K De Cian A Concordet JP Del Bene F. Highly efficient CRISPR/Cas9-mediated knock-in in zebrafish by homology-independent DNA repair. Genome Res. (2014) 24:142–53. doi: 10.1101/gr.161638.113 24179142 PMC3875856

[161] Zhao Y Lin J Wang L Chen B Zhou C Chen T . Derivation and characterization of ovine embryonic stem-like cell lines in semi-defined medium without feeder cells. J Exp Zool A Ecol Genet Physiol. (2011) 315:639–48. doi: 10.1002/jez.715 22021232

[162] Botigelli RC Guiltinan C Arcanjo RB Denicol AC. In vitro gametogenesis from embryonic stem cells in livestock species: recent advances, opportunities, and challenges to overcome. J Anim Sci. (2023)101:skad137. doi: 10.1093/jas/skad137 PMC10199788 37140043

[163] Chen T Zhou J Li M Li Z Feng Y Qiao M . Integrated analysis of lactylation modification and proteomics revealed potential epigenetic regulation in intramuscular fat deposition of Xidu black pigs. BMC Genomics. (2025) 27:86. doi: 10.1186/s12864-025-12428-6 41413458 PMC12831410

[164] Romito A Cobellis G. Pluripotent stem cells: Current understanding and future directions. Stem Cells Int. (2016) 2016:9451492. doi: 10.1155/2016/9451492 26798367 PMC4699068

[165] Li Z Chen L Wu J Chen Y Zhu Y Li G . A review of 3D bioprinting for organoids. Med Rev. (2021) 5:318–38. doi: 10.1515/mr-2024-0089 PMC12362060 40838107

[166] Milner DJ Cameron JA. Muscle repair and regeneration: stem cells, scaffolds, and the contributions of skeletal muscle to amphibian limb regeneration. Curr Top Microbiol Immunol. (2013) 367:133–59. doi: 10.1007/82_2012_292 23224711

